# Conserved HORMA domain-containing protein Hop1 stabilizes interaction between proteins of meiotic DNA break hotspots and chromosome axis

**DOI:** 10.1093/nar/gkz754

**Published:** 2019-09-06

**Authors:** Ryo Kariyazono, Arisa Oda, Takatomi Yamada, Kunihiro Ohta

**Affiliations:** 1 Department of Biological Sciences, Graduate School of Science, The University of Tokyo, 7-3-1 Hongo, Bunkyo-ku, Tokyo, 113-8654, Japan; 2 Department of Life Sciences, The University of Tokyo, 3-8-1 Komaba, Meguro-ku, Tokyo, 153-8902, Japan

## Abstract

HORMA domain-containing proteins such as Hop1 play crucial regulatory roles in various chromosomal functions. Here, we investigated roles of the fission yeast Hop1 in the formation of recombination-initiating meiotic DNA double strand breaks (DSBs). Meiotic DSB formation in fission yeast relies on multiple protein-protein interactions such as the one between the chromosome axial protein Rec10 and the DSB-forming complex subunit Rec15. Chromatin immunoprecipitation sequencing demonstrated that Hop1 is colocalized with both Rec10 and Rec15, and we observed physical interactions of Hop1 to Rec15 and Rec10. These results suggest that Hop1 promotes DSB formation by interacting with both axis components and the DSB-forming complex. We also show that Hop1 binding to DSB hotspots requires Rec15 and Rec10, while Hop1 axis binding requires Rec10 only, suggesting that Hop1 is recruited to the axis via Rec10, and to hotspots by hotspot-bound Rec15. Furthermore, we introduced separation-of-function Rec10 mutations, deficient for interaction with either Rec15 or Hop1. These single mutations and *hop1Δ* conferred only partial defects in meiotic recombination, while the combining the Rec15-binding-deficient *rec10* mutation with *hop1*Δ synergistically reduced meiotic recombination, at least at a model hotspot. Taken together, Hop1 likely functions as a stabilizer for Rec15–Rec10 interaction to promote DSB formation.

## INTRODUCTION

Meiotic recombination is a critical biological process in eukaryotic sexual reproduction, contributing to the proper segregation of pairs of parental homologs into haploid gametes and the increased genetic diversity of the next generation. One of the striking features of this event is that it is triggered by the formation of programmed DNA double strand breaks (DSBs), which are transiently introduced by the conserved meiosis-specific topoisomerase VI-like protein Spo11 (1,2). Also, DSBs are introduced not randomly throughout the genome, but predominantly at discrete sites called DSB hotspots. Therefore, how Spo11 cleaves DNA at hotspots is a critical issue to understand the mechanism of meiotic recombination initiation.

Spo11 has been demonstrated to cooperate with various other proteins to break DNA (referred to as DSB proteins). Previous studies have revealed that DSB proteins can be categorized into several distinct classes in terms of their functions (Table 1), and proteins in the same class can form a complex to generate DSBs: Spo11 and its associated factors such as a TOPOVIB-like protein form a core complex of DSB-formation, while proteins of another category form a regulatory complex whose function is also indispensable for DSB formation. Thus, meiotic recombination is initiated by cooperation of multiple proteins.

**Table 1.** Spo11 complex subunits and axial components in *Saccharomyces cerevisiae*

**Spo11 core complex** (68,69)	Spo11	Ski8	Rec102	Rec104
**RMM complex** (68)	Rec114	Mei4	Mer2	
MRX complex* (70,76,77)	Mre11	Rad50	Xrs2	
Axial Components** (16)	Hop1	Red1	Rec8	

*Essential for DSB formation in *S. cerevisiae*, but not in *Schizosaccharomyces pombe* and *Arabidopsis thaliana*. The mammalian counterpart has not been examined.

**Not essential for DSB formation.

DSB formation is strictly controlled at spatial, temporal, and quantitative levels by various factors (3–6), where chromatin structure plays central roles. At the most fundamental layer, nucleosomes around hotspots directly influence the event. As nucleosomes inhibit the access and function of DSB proteins, they are generally depleted around hotspots. In addition, histone modifications are implicated in recombination initiation. For example, in the budding yeast *Saccharomyces cerevisiae*, DSB hotspots are often found in promoter-overlapping accessible chromatin regions (7–9), around which tri-methylated histone H3K4 (H3K4me3) is enriched (10). Hotspots of the fission yeast *Schizosaccharomyces pombe* are located in accessible chromatin as in budding yeast, but marked with acetylated lysine 9 of histone H3 rather than H3K4me3 (11), and rarely coincided with promoters (12,13). In mice and humans, hotspots are located outside of promoters, but associated with H3K4me3 (14). These characteristics of hotspot-surrounding local chromatin are of pivotal importance for the DSB reaction.

Another important aspect of chromatin-mediated DSB regulation is a high-order chromatin architecture. In this regard, meiotic chromosomes form a unique structure termed ‘axis-loops’, which is composed of the proteinaceous axis, or the axial element (AE), and a number of loops emanating from the AE (15). The AE is implicated in DSB formation, as its components such as meiotic cohesin subunits and other structural proteins are required for DSBs in several organisms including yeasts and mice (16,17). Moreover, in budding and fission yeasts, DSB proteins have been shown to reside in axis sites (18,19). On the other hand, loops are important as they are supposed to contain hotspots. This notion is based on high-throughput sequencing of Spo11-oligo DNA, which is a byproduct of Spo11 DNA cleavage reaction, revealing that DSB sites exhibit anti-correlations with cohesin binding sites (20). Consistently, Spo11 is detected in chromosomal loop regions, although it is initially recruited around pericentromeric and Rec8 binding sites (21). These results suggest that the meiotic axis-loops structure (both the axis and loops) is important for DSB formation.

Axis localization of DSB proteins (except for Spo11) and loop localization of hotspots appear to contradict to each other. Such a discrepancy can be reconciled, if DSB hotspots in loops can transiently interact with the chromosome axis (22). This model is supported by several observations in yeasts in which Spo11 and its partner proteins are localized at both axis sites and DSB hotspots in loops (18–19,23). More importantly, the budding yeast PHD-finger protein Spp1, a subunit of the COMPASS (complex of proteins associated with a trithorax-related SET domain protein) histone H3K4 methyltransferase complex (24,25), can bind to both H3K4me3-marked hotspots and Mer2, an axis-binding DSB protein and facilitate DSB formation probably by mediating interaction between them. However, this model needs to be further verified not only in yeasts but also in other species, since tangible evidence to support it has not been obtained.

Factors for DSB formation appear to be conserved in eukaryotes, at least in terms of their functions (Table 2). In *S. cerevisiae*, the AE components Red1 and Hop1 promotes axis-localization of Mer2, which in turn recruits Rec114 and Mei4, to chromatin to assemble the RMM (Rec114, Mei4, Mer2) complex, a DSB regulatory complex (18). Consistently, Red1 and Hop1 are required for normal levels of meiotic DSB formation (26–28). In mice, IHO1 (functionally similar to Mer2/Rec15) has been shown to form a complex with REC114 and MEI4 (29), and bind to another axial protein HORMAD1 (a mouse Hop1 ortholog) (30). Therefore, the IHO1–REC114–MEI4 complex may be recruited to the axis by HORMAD1. A similar system can work in fission yeast, which lacks the canonical synaptonemal complex (SC) but develops AE-resembling axis structures termed linear elements (31,32). In this yeast, the Spo11 ortholog Rec12 and its binding partners Rec6 and Rec14 form the DSB forming core complex (DSBC) and function with the help of an RMM-analogous regulatory complex consisting of Rec7 (seven), Rec15 (fifteen) and Rec24 (twenty-four) proteins (referred to as the ‘SFT’ complex) (19,33,34). Remarkably, Rec15, the functional counterpart of Mer2 and IHO1 in fission yeast, is localized at both hotspots and the axis sites, and directly interacts with the axis protein Rec10 (fission yeast Red1 ortholog) (19). Thus, fission yeast hotspots may be tethered to the axis through Rec10–Rec15 interaction. Moreover, Hop1 interacts with Rec10 and resides at the axis sites to facilitate DSB formation (35,36). These observations collectively point out an overall similarity of the event among species, but detailed analyses are still necessary for deep understanding of the mechanism.

**Table 2.** Conservation of Mer2 related protein and axial components

*S. cerevisiae*	*S. pombe*	*M. musculus*	*A. thaliana*	Reference
**RMM complex**				
Mer2	Rec15	IHO1	AtPRD3	(30,53)
**Axial Components**				(16,37,54,71,72)
Hop1	Hop1	HORMAD1/2	ASY1/2	(40)
Red1	Rec10	SYCP2	ASY3	(31,64,73,74)
		SYCP3	ASY4	(64)
	Rec25			(35,55)
	Rec27			
Rec8	Rec8	REC8	DIF1	(75,78–81)
		RAD21L		

Here, we focus on the axis-bound Hop1/HORMAD1 protein, as it is required for ‘wild-type’ level DSB formation in multiple species (16,37,38). Hop1 contains a highly conserved HORMA domain (39,40), which is also found in other important chromosomal proteins, such as Rev7 (an accessory subunit of DNA polymerase zeta) and Mad2 (an essential spindle checkpoint protein). HORMA domain-containing proteins are thought to function as regulators with conformational switches for various chromosomal functions (40,41). Despite the expected importance for meiotic recombination, its precise roles in this event have yet to be determined.

In this study, using fission yeast, we conducted chromatin immunoprecipitation (ChIP) followed by quantitative polymerase chain reaction (qPCR) and ChIP-sequencing (ChIP-seq) analyses, protein interaction assays, and point-mutant studies. Our results revealed that Hop1 is localized at hotpots as well as axis sites, which is regulated by Rec10 and Rec15. We also show that Hop1 binds not only to Rec10 but also Rec15, and that Hop1 promotes chromatin-binding of Rec10 and Rec15. Notably, we provide evidence that Hop1 fortifies interaction between Rec10 and Rec15. Based on these results and our previous hypothesis that Rec10–Rec15 binding brings hotspots and axis sites in close proximity, we propose that physical interaction among Hop1, Rec10 and Rec15 facilitates DSB formation to possibly modulate a higher-order chromosome structure.

## MATERIALS AND METHODS

### Yeast strains and plasmid construction

The plasmids and strains used in this paper are listed in Supplementary Tables 1 and 2. Point mutations were introduced by PCR-based site-directed mutagenesis using PrimeSTAR Max (Takara Bio, Shiga, Japan). For the deletion of the *hop1*^+^ gene, PCR was used to construct targeting fragments in which the *bsd^R^* cassette or the *ura4*^+^ cassette were flanked by an upstream and downstream sequences of *hop1*^+^. These targeting fragments were transformed into yeast cells, and *hop1* disruptants were selected on 100 μg/ml of Blasticidin S -containing YES medium (for *bsd^R^*), or uracil-dropout SD minimal medium (for *ura4*^+^). To introduce point mutations in *rec10*^+^ or *hop1*^+^, DNA fragments with corresponding base substitutions were transformed into a strain in which original *rec10*^+^ or *hop1*^+^ loci was disrupted with a *ura4*^+^ marker gene, and strains whose *ura4*^+^ marker was replaced by a mutation-containing fragment were selection on medium containing 800 μg/ml of 5-fluoroorotic acid (5-FOA). Strains expressing epitope-tagged Hop1 were constructed by integration of linearized pNAT plasmids carrying a tagged-version of *hop1*^+^ gene into a locus adjacent to *zfs1^+^* on chromosome 2 into a *hop1* deletion strain. Mutants with correct insertion were selected on YES medium plates containing 100 μg/ml of Nourseothricin/clonNAT.

### Yeast two-hybrid assays and three-hybrid assays

Yeast two-hybrid assays and three-hybrid assays were performed as in previous study (19). *Saccharomyces cerevisiae* AH109 strain (Clontech, Mountain View, CA, USA) were transformed with pGADT7 and pGBKT7 plasmids, respectively, harboring the indicated bait and prey genes and selected on leucine-/tryptophan-dropout SD minimal medium (SD w/o LW). Colonies were streaked on SD w/o LW, and further selected on SD medium without leucine, tryptophan, histidine and adenine (SD w/o LWAH) to assess the interaction between bait and prey proteins. Cells were grown for 5 days at 30°C and their growth was analyzed to assess the interactions between bait and prey proteins.

For yeast three-hybrid assay, *S. cerevisiae* Y190 strain were transformed with pGADT7 and pGBKT7 plasmids and selected on SD w/o LW. Then transformants were sequentially transformed with pADE1 plasmids harboring the ORF of wild-type *hop1* gene or *hop1-9A* under the constitutive budding yeast promoter pTDH3, or the empty pADE1, and selected on SD without leucine, tryptophan and adenine (SD w/o LWA). Colonies were streaked on SD w/o LWA and further selected on SD w/o LWAH + 10mM 3-amino−1,2,4-triazole (3-AT) to assess the interaction among the three proteins. Cells were grown for 5 days at 30°C, and their growth was analyzed on SD w/o LWAH+ 10mM 3-AT to investigate the bridging by Hop1 between bait Rec10 and prey Rec15.

### Culture and synchronized meiotic induction

*Schizosaccharomyces pombe* culture and synchronous induction of meiosis were carried out as previously described (19). Synchronizations of meiotic cell cycle were confirmed by flow cytometry analysis (Supplementary Figure S1).

### ChIP experiments

ChIP assays were conducted as described previously (42) with minor modifications. Cells were fixed as follows: a 40 mL culture at a density of 2 × 10^7^ cells/ml was incubated at room temperature for 15 min after adding 1.1 ml of 37% formaldehyde (final concentration 1%). Fixation was terminated by adding 2.5 ml of 2 M glycine, followed by washing in ice-chilled phosphate-buffered saline, before freezing the cells in liquid nitrogen. For FLAG-tagged Hop1, Rec15 and Rec10, anti-FLAG M2 antibody (SIGMA-Aldrich, St Louis, MO, USA) was incubated with Protein A Dynabeads^®^ (Thermo Fisher Scientific, Waltham, MA, USA) and then added to the cell lysates. For the Rec8 and control IgG ChIP experiments, anti-Rec8 polyclonal antibody (43) and rabbit IgG (Santa-Cruz, Dallas, TX, USA), respectively, were added to cell lysates followed by the incubation and addition of Protein A Sepharose (GE Healthcare, Chicago, IL, USA). After IP, DNA was purified using a FastGene Gel/PCR Extraction Kit (Nippon genetics, Tokyo, Japan). The purified DNA solution was used for qPCR assays and ChIP-sequencing analyses. qPCR was performed with KAPA SYBR FAST qPCR Kit (KAPA Biosystems) and StepOne Real-Time PCR system (Applied Biosystems). The primers used for qPCR are listed in Supplementary Table S3.

### ChIP-sequencing and peak calling

Libraries were prepared with NEBNext ChIP-Seq Library Prep Master Mix Set for Illumina (P/N E6240S, NEB, Ipswich, MA, USA) and NEBNext Multiplex Oligos for Illumina (P/N E7335S, NEB). Multiplexed libraries except for Rec8 were paired-end sequenced by MiSeq (MiSeq Reagent Kit v3; Illumina, San Diego, CA, USA). Libraries for Rec8 IP, control IgG IP and Input for Rec8 IP and IgG IP were single-end sequenced. Reads were mapped to the *S. pombe* 972 *h^−^* genome (AS294v230) using bowtie2. Peak detection was performed with MACS2 callpeak with options of ‘-nomodel –extsize 200 –broad –broad-cutoff 0.05’. For the visualization of each ChIP-sequence data, the IP scores were divided by that of Input, normalized so that the median of log_2_ score is 0 and plotted every 25 bp with 250 bp window size.

### Peak overlaps and analysis on axis and DSB hotspots

Overlaps between called peaks are detected by bedtools coverage with option of ‘-f 0.2’. For further analysis, we designated the 603 hotspots denoted by a previous study in diploid meiosis (44) as ‘DSB hotspots’. We classified Rec10-binding sites into two groups: DSB hotspot-associated Rec10 sites and non-DSB hotspot Rec10 sites. The latter one is Rec10-binding site, <20% of which overlaps one or more DSB sites, and is assumed to be exclusively associated with LinE sites, hence we defined them as ‘axis sites’ (see Supplementary Figure S2D). We excluded centromeric, telomeric and ribosomal DNA region from analysis. The software used is listed in Supplementary Table S4.

### Co-IP cross-linked co-IP

Co-IP and cross-linked Co-IP were conducted as described previously (19). For cross-linked Co-IP, cells were fixed and frozen as described in the ‘ChIP experiments’ section.

### Statistical analyses

For comparison among two groups, two-sided Welch's *t*-test was applied. Statistical significance ware indicated as *P < 0.05 and **P < 0.01.

### Prediction of protein structure

The Rec10 N-terminal (201–400) protein structure was predicted using the PHYRE2 Protein Fold Recognition server (Protein Homology/analogY Recognition Engine V 2.0;http://www.sbg.bio.ic.ac.uk/phyre2/) (45). For details, see Supplementary Data.

Secondary structure predictions of Rec10 orthologs were obtained by using JPred4 (http://www.compbio.dundee.ac.uk/jpred/) (46).

### Pulsed field gel electrophoresis (PFGE) and Southern blotting

Pulsed field gel electrophoresis (PFGE) experiments were conducted as described previously (47). For electrophoresis, DNA embedded in plugs was separated in a 0.8% agarose (pulsed-field certified, BioRad, Hercules, California) gel in 1 × TAE (Tris 40 mM, acetic acid 40 mM, ethylenediaminetetraacetic acid 1 mM) at 14°C for 48 h. Conditions for PFGE with CHEF Mapper (BioRad) were as follows: included angle, 106°; initial switching time, 20 min; final switching time, 30 min. Gels were stained with × 10 000 diluted SYBR^®^ GreenI (Lonza, Basel, Switzerland) for 1 h and the gel image was captured by ImageQuant LAS 4000 (GE Healthcare).

### Recombination assay

Mating and sporulation of haploid cells were conducted as previously described with minor modifications (48). After a two-days incubation period on SPA at 30°C, cells were digested in 0.5% glusulase (PerkinElmer, Waltham, MA, USA) for 3 h to isolate spores. A total of 1000 spores were inoculated on YEA plates and grown at 30°C for 3 days, and spore viability was estimated by calculating (number of colonies grown on YEA)/1000. To measure *ade6-M26* x *ade6-469* intra-genic recombination frequency, 1 × 10^4^ spores were inoculated on YEA plates followed by incubation at 30°C for 2 days so that actual numbers of viable colony were [spore viability × 10 000], then replica-plated on EMM2 without adenine (EMM2-A). The intra-genic recombination frequency of *ade6* was calculated as (number of colonies grown on EMM2-A)/(spore viability × 10 000).

### Protein extraction and immunoblotting analysis

Protein was extracted as previously described (19). Anti-FLAG M2 antibody (Sigma-Aldrich) and anti-HA 3F10 antibody (Sigma-Aldrich) were used as the primary antibodies at a dilution ratio of 1:2000, and anti-mouse IgG-HRP (GE Healthcare) and anti-rat IgG-HRP SC-2006 (Santa-Cruz) were used as the secondary antibodies at 1:2000 dilution.

### Microscopic observation

Haploid cells were grown on YEA plates overnight at 30°C and then spotted on SPA plates. After incubating for 6 h at 30°C, the spots were fixed with methanol overnight at −20°C and then stained with 1 μg/ml of 4′,6-diamidino-2-phenylindole (DAPI). Cells were re-suspended and washed with PEMS (100 mM PIPES pH 7, 10 mM MgSO_4_, 1 mM EGTA, 1.2 M sorbitol) and observed by fluorescent microscopy.

## RESULTS

### Colocalization of Hop1 with Rec15 and Rec10 in the axis and DSB hotspots

**Figure 1.**
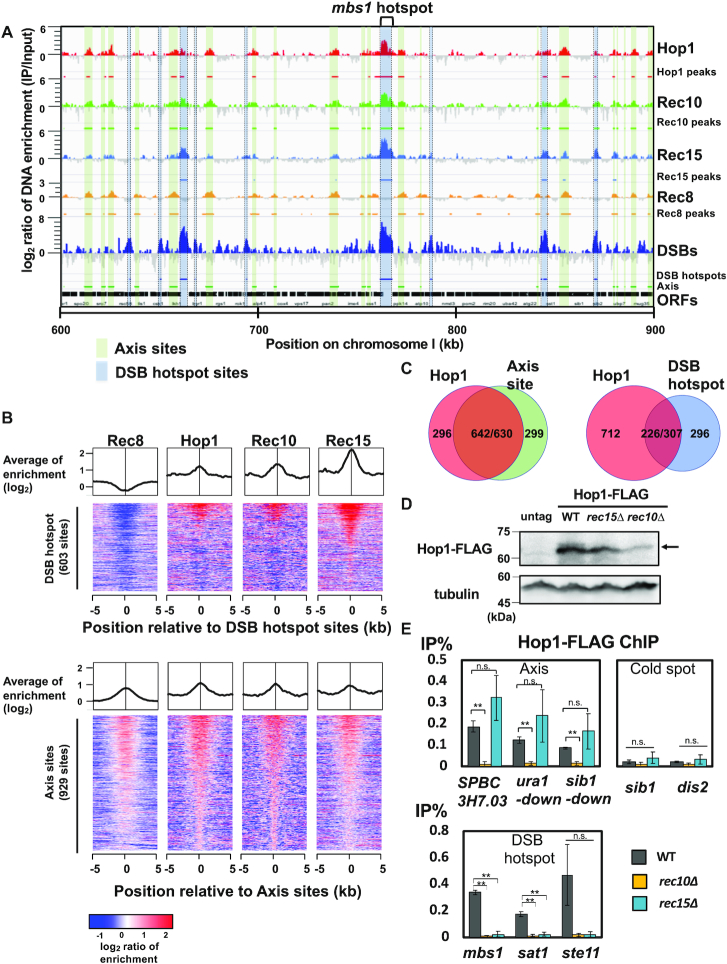
Genome-wide analysis reveals Hop1 localization in DSB hotspots and the chromosomal axis. (**A**) ChIP-seq data of Hop1, Rec10, Rec15 and Rec8 around a *mbs1* DSB hotspot locus (KR306, OTM595, OTM416 and YZ980, respectively). Cells were cross-linked and harvested at 4 h after meiotic induction (for Hop1, Rec15 and Rec10 ChIP-seq) and at 3.5 h (for Rec8 ChIP-seq). The *Y*-axis represents log_2_ scaled DNA enrichment (IP-read counts per Input-read counts). Black horizontal bars represent the open reading frames. Bands with blue shading between two dotted lines indicates the region of the *mbs1* DSB hotspot and other hotspots. Light green-shaded bands represent the regions of axis (LinE) sites. The DSB hotspot positions data and lane of DSBs represents the count of Rec12 oligonucleotides in diploid meiosis described in previous study (44). (**B**) Averaged binding signals and heat map images of ChIP-seq signals of Rec8, Hop1, Rec10 and Rec15 around the 603 DSB hotspots (top) and the 929 axis sites(bottom). In heat map images, DSB hotspots are sorted by Rec12-oligo counts reported in the previous study (44). Axis sites are sorted by Rec10 ChIP fold enrichments. (**C**) Venn-diagrams showing the overlap between Hop1 sites and axis sites (left), and between Hop1 sites and DSB hotspots (right). The numbers indicate the number of binding sites. In the left Venn-diagram, two numbers separated by ‘ / ’ in the intersection region (642/630) indicates 642 Hop1 peaks overlap with 630 axis sites. The discrepancy of peak count in intersection occurs because occasionally more than one Hop1 sites overlaps one axis site. Likewise, in the right diagram (226/307) indicates 226 Hop1 peaks overlap with 307 DSB hotspot sites. (**D**) Protein abundance of FLAG-tagged Hop1 in wild-type, *rec15Δ* and *rec10Δ* cells (KR306, KR310 and KR308, respectively; KR98 was used as untagged control). Upper picture: anti-FLAG blotting. Lower picture: anti-tubulin blotting. An arrow indicates the position of Hop1-FLAG. Cells were harvested at 4 h after meiotic induction. (**E**) ChIP-qPCR analysis of Hop1 in the background of wild-type, *rec10Δ* and *rec15Δ* (KR306, KR308 and KR310, respectively). Error-bars represent standard deviations (S.D.) (*n* = 3). q-PCR experiments were conducted with the primer sets for the axis sites (axis; *SPBC3H7.03c, ura1-down, sib1-down*), DSB hotspot (DSB; *mbs1, sat1* and *ste11*), and cold spot sites (cold spot; *sib1* and *dis2*) (see Supplementary Figure S3). Asterisks * and ** represent a statistical significance at <5% and <1% by two-sided Welch's *t*-test, and n.s. indicates not significance.

To gain insights into the function of Hop1, we first investigated the genome-wide distribution of fission yeast Hop1 by ChIP-seq analysis, in comparison with the DSB hotspots and axis (Figure 1A and Supplementary Figure S2A). The positions of DSB hotspots were defined according to the previous genome-wide analyses of Rec12-oligo by Smith's group (44) which created the most precise DSB map. Although the DSB map was obtained on diploid meiotic cells and our genome-wide analyses were performed on haploid ones, we note that an early study reported that the positions and amounts of DSBs around *mbs1* and *mbs2* hotspots are similar in diploid and haploid cells (49). The counterpart of the axis in fission yeast is Linear elements (LinEs), whose major component is Rec10 (50). Previous studies had defined the genomic region of linear elements, as Rec10 binding region outside of DSB hotspots (19,51). We redefined 929 non-DSB Rec10 sites (axis sites) based on our ChIP-seq data of Rec10 (Supplementary Figure S2B–D, and see *Peak overlaps and analysis on axis and DSB hotspots* in ‘Materials and Methods’ section). Around these axis sites, Rec8 cohesin are enriched (Figure 1B, left and Supplementary Figure S2E, left), consistent with the previous notion that axis sites are enriched with Rec8 cohesin. We detected only a few overlaps of Rec8 peaks and DSB hotspots (Supplementary Figure S2E, right) as reported previously (52): only 32 of the 999 Rec8 peaks overlapped with DSB hotspots.

We detected 938 Hop1 peaks on chromosome arms and compared them to the axis sites and the Rec12 oligo peaks (‘DSB hotspots’). We observed that the majority of Hop1 peaks are colocalized with the axis sites (642 of 938 Hop1 peaks, 68.4%; Figure 1C, left and Supplementary Figure S2F), indicating that Hop1 is mainly localized in the axis (Figure 1B), consistent with the finding that 823 Hop1 peaks (87.7%) overlapped with the Rec10 binding sites (Supplementary Figure S2G, left).

On the other hand, 226 of 938 Hop1 peaks (24.0%) overlapped with DSB hotspots (Figure 1C, right). It should be noted that more than half of the Hop1 sites (549 of 938 Hop1 peaks, 58.5%) coexisted with Rec15 binding sites (Supplementary Figure S2G, center).

The binding of Hop1 to both the axis and DSB hotspots was similar to those of Rec10 and Rec15 reported in our previous study (19) (Figure 1B and C; Supplementary Figure S2H). We therefore speculated that Hop1 may interact with Rec10 and Rec15. As such, we examined whether Hop1 distribution is affected in the absence of Rec10 or Rec15 by qPCR-based ChIP assay (Figure 1D and E and the position of primers are shown in Supplementary Figure S3). In the *rec15Δ* strain, the Hop1 protein level was comparable to wild-type cells, and Hop1 was retained in the axis sites, but was no longer detected in the DSB hotspots (Figure 1E). In *rec10*Δ, Hop1 localization was lost on both the axes and DSB hotspots, although such phenotype may be partly caused by reduced protein amount or stability (Figure 1D). Accordingly, Rec10 is a prerequisite for chromatin-binding of Hop1, and Rec15 is required for the DSB hotspot-binding of Hop1. This suggests a model that Hop1, which can interact with both Rec15 and Rec10, is stabilized and loaded to the axis by Rec10, and recruited to hotspots by DSB hotspot-associated Rec15. Consistent with this idea, Hop1 interacts with Rec10 (35,36), and mouse Hop1 homolog HORMAD1 interacts with IHO1, a functional counterpart of Rec15 in mouse (30).

### Hop1 interacts with Rec15 and Rec10

**Figure 2.**
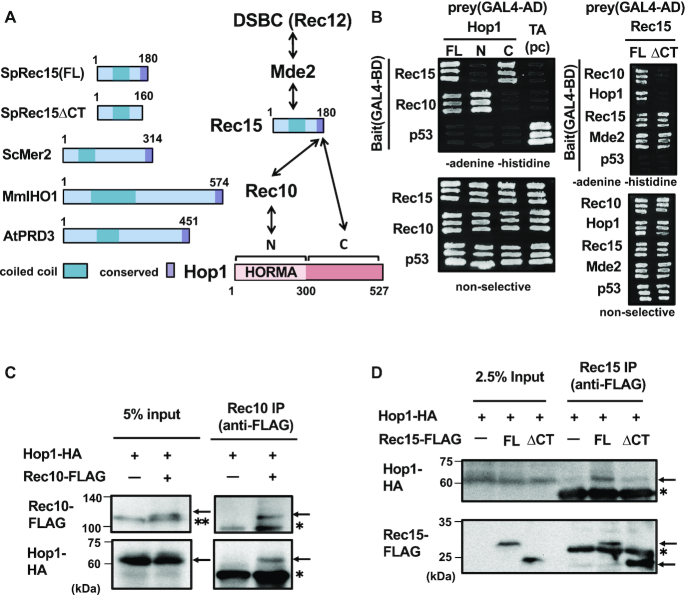
Hop1 interacts with both Spo11 complex protein Rec15 and axial protein Rec10. (**A**) (Left) Conservation of domains in Rec15 related proteins. Sp: *Schizosaccharomyces pombe*, Sc: *Saccharomyces cerevisiae*, Mm: *Mus musculus*, At: *Arabidopsis thaliana*. Coiled coil and conserved C-terminus domains are indicated in light blue and purple boxes, respectively. (Right) Summary of interactions between Hop1, Rec10, Rec15 and DSBC complex. Hop1 binds to Rec10 with its N-terminus domain and to Rec15 with its C-terminus domain. Rec15 binds to both Hop1 and Rec10 with its conserved C-terminus domain. (**B**) Y2H assay between Hop1 and Rec10 or Rec15. Hop1 were divided into N (1–300) and C (301–527) fragments (see panel A). Rec15 full-length (FL, 1–180) and ΔCT (1–160) were also tested. T-antigen (TA) and p53 were used as a positive control (pc) set. Interaction between bait and prey proteins allow cells to grow in the absence of adenine and histidine. (**C**) Co-IP experiment between Rec10-FLAG and Hop1-HA. Cells were harvested at 4 h after meiotic induction. The upper arrows indicate the position of Rec10-FLAG and the lower arrow indicates the position of Hop1-HA. Single asterisks indicate the position of immunoglobulins and a double asterisk indicates non-specific bands. The numbers in the left represent the molecular weight standards in kDa. (**D**) Co-IP experiment between Rec15-FLAG and Hop1-HA. Cells were treated with a crosslinker (1% formaldehyde) at 4 h after meiotic induction. Anti-FLAG IP was conducted after micrococcal nuclease (MNase) treatment. The upper arrow indicates the position of Hop1-HA; the middle and lower arrows indicate the positions of Rec15-FLAG and Rec15ΔCT-FLAG, respectively. An asterisk indicates the position of immunoglobulins.

We then examined the interaction of Hop1 with Rec15 and Rec10 by yeast two-hybrid (Y2H) assays followed by domain analyses (Figure 2A and B). The Y2H assay results confirmed that the full-length and N-terminal region of Hop1 (harboring HORMA domain) interact with Rec10, and that the C-terminus of Hop1 interacts with Rec15 (Figure 2B, left).

We also explored which Rec15 domains are required for its interaction with Rec10, Hop1 and Mde2, as well as with Rec15 itself. Mde2 is another essential meiotic DSB protein which interacts with the Rec7-Rec15-Rec24 (SFT) and Rec12-containing (DSBC) subcomplexes (19) (Figure 2A). It stabilizes the SFT subcomplex and recruits the DSBC subcomplex to DSB sites, after DNA replication. Deletion of the conserved C-terminal segment of Rec15 (53) (Rec15ΔCT, Figure 2A) led to a marked reduction in its interaction with both Hop1 and Rec10, though Rec15ΔCT was capable of binding to Mde2 and Rec15–Rec15 self-interaction (Figure 2B, right). Therefore, the Rec15 C-terminal domain functions as a common binding domain for Hop1 and Rec10, but not for Mde2 or Rec15 itself.

To confirm binding of Hop1 to Rec10 and Rec15 *in viv*o, interactions between Hop1-HA and Rec15-FLAG (full-length and ΔCT) or Rec10-FLAG was tested by co-IP experiments. We detected Hop1-HA in the Rec10-FLAG immunoprecipitates (Figure 2C, bands indicated with arrows), which means that Hop1 and Rec10 interact with each other. The interaction between the full-length Hop1-HA and Rec15-FLAG was also detected by co-IP experiments using a crosslinked condition (Figure 2D), supporting the existence of an interaction between Hop1 and Rec15. We also tested whether the C-terminus-deleted Rec15-FLAG protein (Rec15ΔCT-FLAG) forms a complex with Hop1-HA by co-IP experiments, and found that Hop1-HA was barely recovered in immunoprecipitates with Rec15ΔCT-FLAG (Figure 2D). These results suggest that the C-terminal domain of Rec15 is involved in interaction with Hop1. Since we previously reported that Rec15 interacts directly with Rec10 (19), Hop1 can bind to both of Rec10 and Rec15, implicating that such ternary physical interaction might be a basis of hotspot- and axis-association of Hop1.

### Hop1 C-terminal domain serves as a binding interface for Rec15

To verify the possibility that Hop1 is recruited to hotspots by Rec15, we wished to examine a possible contribution of the Hop1–Rec15 interaction. In this regard, C-terminus of Hop1, essential for Rec15 binding, carries a PHD-finger domain, and we speculated that this domain may serve as an interface for Rec15 binding. We hence applied alanine scanning to the domain and assessed Hop1-Rec15 association by Y2H analyses. This assay led us to substitute nine residues in the Hop1 PHD-finger to alanine (*hop1-9A*). These mutations destroyed its interaction with Rec15, but retained the binding ability to Rec10 (Supplementary Figure S4A–C). ChIP-qPCR analyses revealed that Hop1-9A enrichment is reduced at *mbs1* DSB hotspot site, but not for *sat1* and *ste11* hotspot loci, while axis-bound Hop1-9A protein levels are comparable to wild-type Hop1 (Supplementary Figure S4D). These data indicate that Hop1 is recruited to DSB hotspots through the interaction between Hop1 and Rec15, which is similar to previously reported Rec15-dependent hotspot localization of Rec10 (19).

### Rec10 localization on DSB hotspot depends on Hop1

**Figure 3.**
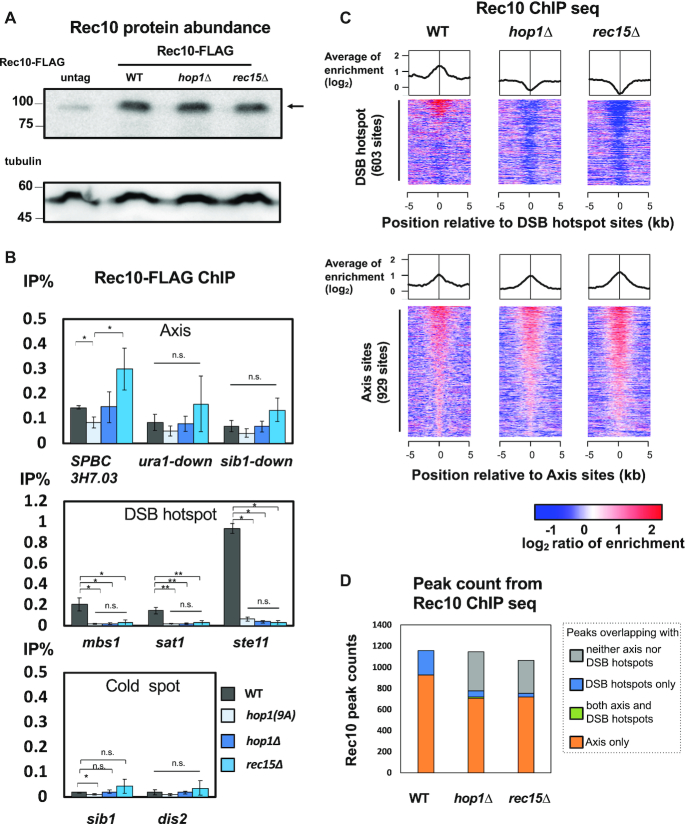
Hop1 influence on the localization of Rec10. (**A**) Protein abundance of FLAG-tagged Rec10 in wild-type, *hop1Δ*, and *rec15Δ*. An arrow indicates the position of Rec10-FLAG (OTM595, KR39 and OTM607, respectively. OTM557 was used as untagged control). Upper picture: anti-FLAG blotting. A faint band in the untagged lane is a non-specific band. An arrow indicates the position of Rec10-FLAG. Lower picture: anti-tubulin blotting. (**B**) The enrichment of Rec10-FLAG on the axis, hotspot and cold spot sites in wild-type, *hop1–9A, hop1Δ* and *rec15Δ* (OTM595, KR144, KR39 and OTM607, respectively). Error-bars represent standard deviations (S.D.) (*n* = 3). A ChIP-qPCR assay was performed as in Figure 1D. Asterisks * and ** represent a statistical significance at <5% and <1% by two-sided Welch's *t*-test, respectively. n.s. indicates not significant. (**C**) Averaged binding signals and heat map images of ChIP-aeq signals of Rec10 around DSB hotspot sites and axis sites in wild-type, *hop1Δ* and *rec15Δ*. Heatmaps are displayed as in Figure 1B. ChIP-seq experiments were conducted as in Figure 1A. (**D**) Stacked bar graphs of numbers of Rec10 peaks in wild-type, *hop1Δ* and *rec15Δ*. Called peaks were classified according to their overlaps with axis sites or DSB hotspots.

To further understand the significance of interaction among Hop1, Rec10 and Rec15, we next investigated how Hop1, Rec15 and their interaction influence chromatin binding of Rec10. The steady state protein level of Rec10 was not affected by lack of Hop1 or Rec15 (Figure 3A). ChIP-qPCR experiments revealed that Rec10-FLAG enrichment in the axis was not significantly affected in *hop1Δ* (Figure 3B top), but interestingly, the binding of Rec10-FLAG on DSB hotspots was markedly reduced by *hop1* deletion (Figure 3B, middle).

This effects of *hop1*Δ on Rec10 localization on DSB hotspots is similar to that of *rec15*Δ, which was previously reported (19) (Figure 3B). These effects were confirmed by genome-wide ChIP-seq analysis: Rec10 localization on DSB hotspots is deteriorated in *hop1*Δ and abolished in *rec15*Δ (Supplementary Figure S5A; Figure 3C and D). Overall, Hop1 and Rec15 promoted localization of Rec10 at hotspots, but not at axis sites. Notably, in *hop1-9A* mutants, DSB hotspot-bound Rec10-FLAG was reduced, as observed in *hop1Δ* (Figure 3B). Since Hop1-9A is defective in its interaction with Rec15 but is still able to interact with Rec10, Hop1–Rec15 interaction would assist Rec10 recruitment to the DSB hotspot.

### Hop1 enhances Rec15 localization on both DSB hotspot and axis

**Figure 4.**
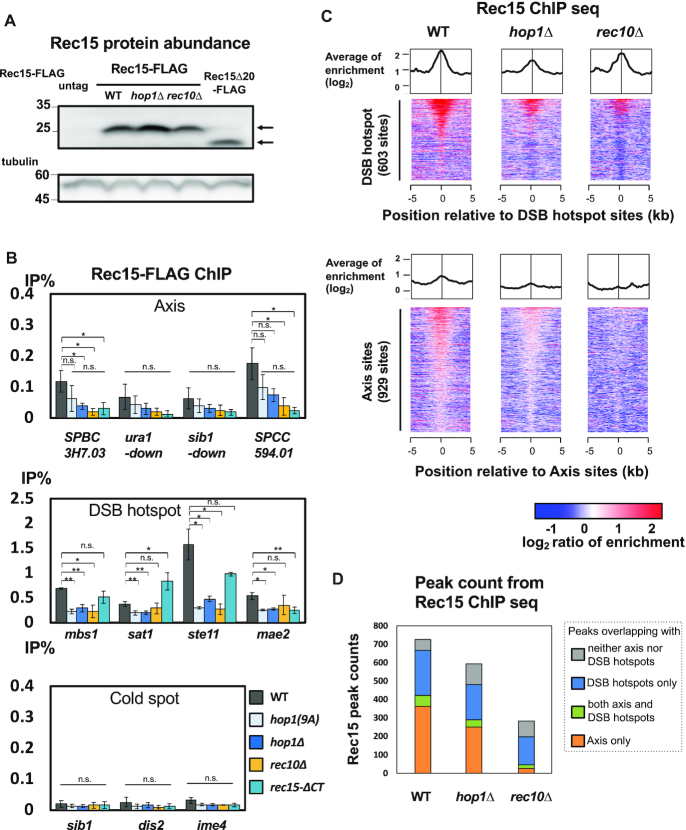
Hop1 influence on the localization of Rec15. (**A**) Protein abundance of FLAG-tagged Rec15 in wild-type, *hop1Δ, rec10Δ* background and FLAG-tagged Rec15ΔCT (OTM416, KR38, OTM578 and KR95, respectively. OTM557 was used as untagged control). Upper picture: anti-FLAG blotting. An upper arrow indicates the position of Rec15-FLAG and a lower arrow indicates the position of Rec15ΔCT-FLAG protein. Lower picture: anti-tubulin blotting. (**B**) Enrichment of Rec15-FLAG on axis, hotspot and cold spot sites in wild-type, *hop1-9A, hop1Δ, rec15ΔCT-FLAG* and *rec10Δ* (OTM416, KR143, KR38, OTM578 and KR95, respectively). Error bars represent the S.D. of three biological replicates. q-PCR experiments were conducted with the primer sets for the axis sites (axis; *SPBC3H7.03c, ura1-down, sib1-down, SPCC594.01*), DSB hotspot (DSB; *mbs1, sat1, mae2 and ste11*), and cold spot sites (cold spot; *sib1, dis2* and *ime4*) (see Supplementary Figure S3). Asterisks * and ** represent a statistical significance at <5% and <1% by two-sided Welch's *t*-test, and n.s. indicates not significant, respectively. (**C**) Averaged binding signals and heat map images of ChIP-aeq signals of Rec15 around DSB hotspot sites and axis sites in wild-type, *hop1Δ* and *rec10Δ*. ChIP-seq experiments were conducted as in Figure 1A. Heatmaps are displayed as in Figure 1B. (**D**) Stacked bar graphs of numbers of Rec15 peaks in wild-type, *hop1Δ* and *rec10Δ*. Called peaks were classified according to their overlaps with axis sites or DSB hotspots.

Having shown that Rec10 requires Hop1 and Rec15 for its hotspot-localization, we next examined how Rec15 localization is affected by Hop1 and Rec10. Rec15 protein abundance in *hop1*Δ was comparable to the wild-type (Figure 4A). In *hop1*Δ mutants, Rec15 localization was in general modestly reduced (by 30–50%) both on axis and DSB hotspot sites, and similar was the case with *hop1-9A* mutants (Figure 4B). Although the degree of the reduction in ChIP-qPCR data were variable depending on chromosomal locations, genome wide tendency of Rec15 localization on axis is modestly reduced in *hop1*Δ, while *rec10*Δ almost completely eliminated axial localization of Rec15 (Supplementary Figure S5B, axis sites indicated with shaded green bands, Figure 4C and D). These results provide the following two insights into chromatin binding of Rec15. First, DSB binding of Rec15 is partially dependent on Hop1 and Rec10. Second, axis binding of Rec15 also requires Hop1 to some extent, but more severely Rec10.

We also examined the axis and hotspot binding of the *rec15ΔCT* mutant. In this mutant, as shown in Figure 2B, the interactions of Rec15 with Hop1 and Rec10 were markedly reduced. ChIP-qPCR experiments revealed that Rec15ΔCT is localized to DSB hotspots at wild-type level (Figure 4B, middle). However, its axis localization was markedly weakened, as previously observed in *rec10Δ* mutants (Figure 4B, top) (19). Therefore, Rec15 would bind to hotspots even without its C-terminal domain, but its localization to the axis sites requires binding to Hop1 and Rec10 through its C-terminal domain. Taking results shown in Figures 3 and 4 into account, the bindings of Rec15 to both the axis and DSB hotspots are promoted by Hop1, which likely allows increased access of Rec10 to DSB hotspots (see Figures 3B and 4B).

### The interaction between Hop1 and Rec15 enhances DSB formation

We then asked whether Hop1–Rec15 interaction contributes to meiotic recombination. To this end, testing *hop1-9A* mutants, which was deficient at Rec15 binding, were tested for DSB formation and the recombination frequency. We observed that the *hop1-9A* mutation reduces DSB formation, to a similar level with *hop1*Δ cells (Supplementary Figure S6A–D). Consistently, we detected a substantial reduction of intra-genic recombination frequency between the *ade6-M26* and *ade6-469* alleles in *hop1-9A* and *hop1Δ* (Supplementary Figure S6E). Since the two mutants exhibited similar levels of defects, Hop1 would need to interact with Rec15 to promote meiotic recombination.

### Hop1 and Rec10 redundantly recruit Rec15 onto the axis

**Figure 5.**
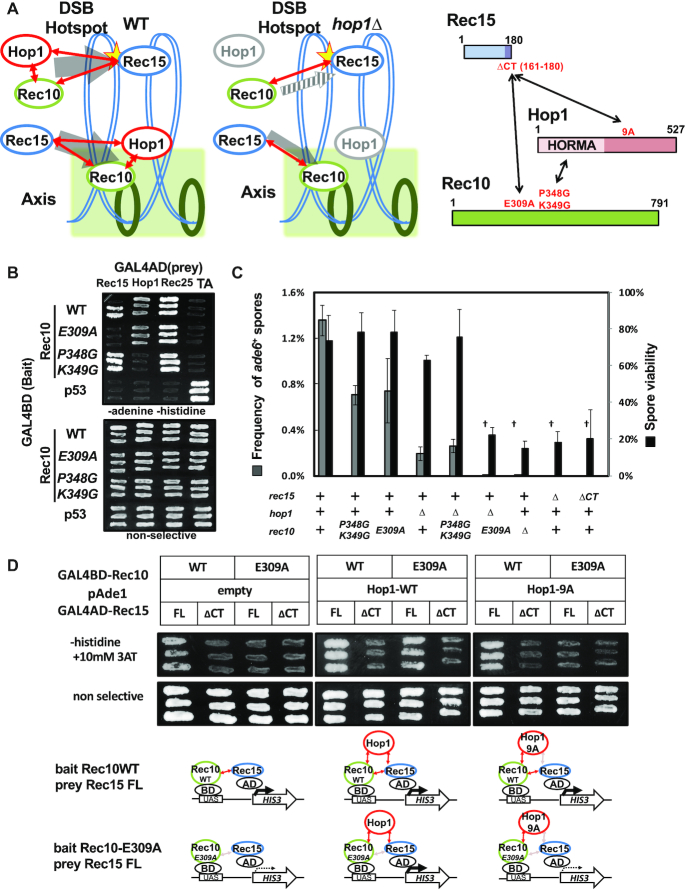
Redundant pathways for recruitment of DSB machinery to the axis. (**A**) Schematic diagram of redundant pathways of Rec15 recruitment to axis by Rec10 in the presence (left) or absence (middle) of Hop1. Even in absence of Hop1, reduced amount of Rec15 localizes on axis (gray arrow) via interaction between Rec10 and Rec15. (right) Interactions between Hop1, Rec10 and Rec15. Mutants deficient in each interaction are described in red. (**B**) Y2H assay between Rec10 mutants versus Rec15, Hop1 and Rec25. (**C**) Synthetic recombination and sporulation defect in *rec10-E309A* and *hop1Δ*. Intra-genic recombination frequency at *ade6* (gray bars, scale indicated in left *Y*-axis) and spore viability (black bars, scale indicated in right *Y*-axis) were measured. Error bars represent S.D. of several biological replicates (five in wild-type, three in *rec10P348G K349G, rec10-E309A, hop1Δ* and *hop1Δ rec10P348G K349G*, four in *hop1Δ rec10-E309A, rec10Δ* and *rec15Δ*, and three in *rec15ΔCT*). Daggers (†) indicate that two Ade^+^ colonies were generated in total 2.68 × 10^4^ spores (in *hop1Δ rec10-E309A* double mutant), 1.96 × 10^4^ spores (in *rec10*Δ mutant) and no Ade^+^ colony was generated in 2.36 × 10^4^ spores (in *rec15*Δ mutant) and 3.26 × 10^4^ spores (in *rec15ΔCT* mutant), respectively. (**D**) Yeast three-hybrid experiments indicating tripartite interaction among Rec10, Rec15 and Hop1. Efficient interaction among the three proteins activates transcription of the reporter gene *HIS3*, whose product enables cells to grow on histidine-lacking and 3-AT (an inhibitor of His3p)-containing plates (left) Rec10 and Rec15 interact directly and Rec10*-*E309A mutant lost ability to bind Rec15. (middle) Expression of Hop1 restored interaction between Rec15 and Rec10*-*E309A, which indicates that Hop1 mediates interaction between Rec10 and Rec15. (right) Expression of Hop1-9A which is deficient in Rec15 binding did not restore interaction between Rec15 and Rec10*-*E309A, which indicates that mediator function of Hop1 depends on its ability to bind to Rec15.

Results so far indicate that Hop1 plays substantial roles in DSB formation together with Rec10 and Rec15. However, *hop1*Δ causes less defects compared with *rec10Δ* or *rec15Δ*. In the absence of Hop1, reduced but still a detectable amount of Rec15 localize on DSB hotspot and axis, while *rec10*Δ causes almost complete loss of axial localization of Rec15 (Figure 4B and C). DSB formation is reduced but retained detectable level (Supplementary Figure S6A) in *hop1Δ*, while *rec10*Δ causes complete loss of DSB formation (54). One of the possible explanations for distinct phenotypes between *hop1Δ* and *rec10*Δ*/rec15*Δ would be that Hop1 may enhance direct binding between Rec10 and Rec15 (19) (Figure 5A).

To explore this notion, we isolated new point mutants of Rec10 with reduced ability to interact with either Rec15 (*rec10-E309A*) or Hop1 (*rec10-P348G K349G*) (Figure 5B), by combining detailed Y2H-based domain analysis, prediction of secondary structure of Rec10 and motif searching (Supplementary Figure S7A–C and Supplementary Data). These two mutant proteins retain ability to interact with Rec25, which is an interactor of Rec10 and essential for proper chromosomal localization of Rec10 (36,55) (Figure 5B). Furthermore, both mutant proteins exhibited nuclear localization comparable to wild-type proteins, which is indicative of their localization in the axis (Supplementary Figure S7D), and the amount of protein was comparable to the wild-type Rec10 (Supplementary Figure S7E). However, consistent with the Y2H results, co-IP experiments revealed that Rec10-P348G K349G, but not Rec10-E309A, shows reduced binding to Hop1 (Supplementary Figure S7E, arrowhead).

*rec10-E309A* and *rec10-P348G K349G* mutants exhibited a moderate reduction of intra-genic recombination frequency between the *ade6-M26* and *ade6-469* alleles, compared to *hop1Δ* (Figure 5C). When the meiotic recombination frequency in a double mutant for *hop1Δ rec10-P348G K349G* was examined, we observed very similar levels of reduction in recombination frequency as in the *hop1Δ* single mutant. These results imply that major, if not all, roles of Hop1 in recombination would be mediated by binding to Rec10.

Interestingly, the *hop1Δ* and *rec10-E309A* double mutant showed undetectable levels of recombination frequency and very low spore viability (Figure 5C). In line with these observations, pulsed field gel electrophoresis analysis revealed a marked reduction of DSB frequency in the *hop1Δ rec10-E309* double mutant (Supplementary Figure S8), to almost similar levels in the *rec10Δ* mutant. This finding indicates that Hop1 is more strictly required for DSB formation when interaction between Rec10 and Rec15 is compromised. It is consistent with the idea that Hop1 enhances Rec10–Rec15 interaction.

To further substantiate the idea, the yeast three-hybrid was performed (Figure 5D). As expected from Miyoshi *et al.* (19), growth on selective media revealed that wild-type Rec10 and wild-type Rec15 directly bind to each other even without Hop1. This interaction was not observed between wild-type Rec10 and Rec15ΔCT or between Rec10*-*E309A and wild-type Rec15. Remarkably, presence of Hop1 significantly improved the growth of yeast cells expressing Rec10-E309A and wild-type Rec15, indicating that Rec10*-*E309A, Hop1 and Rec15 form a complex. It is of note that such restoration was not observed with Hop1-9A, which is deficient at binding to Rec15, nor with Rec15ΔCT, which lacks a domain for Rec10/Hop1 binding. These results collectively suggest that Hop1 would promote Rec10-Rec15 interaction to facilitate meiotic recombination initiation.

## DISCUSSION

### Hop1 is involved in preparing meiotic chromosomes for DSB formation

This study revealed that the fission yeast axis protein Hop1 promotes interaction of the axial protein Rec10 (corresponding to Red1 in budding yeast) to DSB hotspots, via its interaction with both Rec10 and Rec15 (a member of the DSB-forming complex; functional counterparts in budding yeast and mammals are Mer2 and IHO1, respectively). These results, along with previous studies, raise possible roles of Hop1 in establishing DSB-competent meiotic chromosomes.

After DNA replication, chromosome axis sites are sequentially bound by cohesin subunits such as Rec8 and Rec11, which in turn recruit Rec10, then Hop1 and at last Rec15. This idea is supported by the following four observations: (i) the axial localization of Rec10 is severely affected by the deletion of Rec8 and Rec11 (19,31); (ii) in the Rec10 deletion, the axis localization of Hop1 disappears (see Figure 1E) and has a strong effect on DSB formation and recombination frequency; (iii) in the *hop1* deletion strain, the axial localization of Rec10 is only slightly affected (Figure 3B); (iv) Rec15 is required for axis binding of neither Rec10 (Figure 3B) nor Hop1 (Figure 1E). Such hierarchy is consistent with the previous observations of budding yeast (18,56) and the observation of fission yeast that the *rec10* deletion confers a stronger phenotype for recombination and DSB formation than mutations of the axial factor Hop1 (54,57).

The present study also demonstrates that Hop1 and Rec10 promote hotspot-binding of Rec15. However, since a substantial amount of Rec15 was detected at hotspots even in the absence of Hop1 and Rec10, and the C-terminal domain of Rec15, which we showed to be responsible for interaction with Hop1 and Rec10, was dispensable for the hotspot binding, Rec15 would have a capability to be localized at hotspots without assistance of Rec10 and Hop1 (see Figure 4).

In this scenario, Hop1 may have important regulatory roles like other HORMA domain proteins such as Mad2, because its absence reduced Rec10 levels at hotspots and modestly decreased Rec15 levels at axis sites and hotspots. Although deletion of the *hop1^+^* gene has milder defects than that of *rec10^+^* and *rec15^+^*, we believe dissecting Hop1’s roles is informative to understand the regulation of meiotic recombination initiation.

### Possible conservation of axial components, the DSB forming complex and their interactions

In several species including budding yeast and mice, it is proposed that loops, in which hotspots are located, are transiently brought to the chromosome axis, with which many DSB proteins are enriched and hotspot-surrounding DNA is cleaved by the fully-assembled DSB-generating machinery. This model needs to be further verified, particularly in fission yeast, as the AE in fission yeast does not maturate into an SC (31,32). Nevertheless, previous findings from our group are consistent with it. Since Rec10 (Rec10 is localized at the axis without Rec15) and Rec15 (a fraction of Rec15 is localized at hotspots without Rec10) can directly interact with each other, interaction between Rec10 and Rec15 might connect hotspots and axis sites (19).

In terms of this model in fission yeast, our present study provides intriguing results that Hop1 could contribute to interaction between axis and hotspots: In spite of weakened association of Rec10 and Rec15, substantial amount of DSBs (Supplementary Figure S8C) and recombinants (Figure 5C) were formed. This observation suggests that axis-loops interaction may be maintained by a pathway mediated by Hop1. Several results further support this notion. (i) In the *rec10-E309A* mutation, lacking Rec10-Rec15 interaction, meiotic recombination frequency decreases to about 50% of the wild-type and the overall DSB level is only limitedly affected. Remarkably, however, combining *hop1Δ* with *rec10-E309A* leads to the complete abolishment of meiotic recombination and DSB formation. Therefore, Hop1 would compensate for robust Rec10-Rec15 interaction to maintain meiotic recombination. (ii) The *rec10-P348G K349G* mutations, which results in a deficient Rec10–Hop1 interaction, does not aggravate partial recombination defects of *hop1Δ* mutants (Figure 5C and Supplementary Figure S8C), indicating that functions of Hop1 are likely to be mediated by Rec10-Hop1 interaction. (iii) Introduction of the *hop1-9A* mutation, which diminishes the binding of Hop1 to Rec15, but not Rec10 and axis-localization (see Supplementary Figure S4), leads to substantial defects in DSB formation, recombination frequency and axis-binding of Rec15, as observed in the *hop1*Δ mutant. Taken together, Hop1 and interaction among Hop1, Rec10 and Red15 would play critical roles in meiotic DSB formation, probably through axis-loop interaction. It is also of note that Hop1 serves as a potential molecular matchmaker to promote the interaction between Rec10 and Rec15. Although roles of Hop1 as a molecular matchmaker may be eclipsed by direct interaction between Rec10 and Rec15, it certainly plays an important regulatory function to activate DSB hotspots.

### Other aspects of Hop1 as an axial protein

Rec 10, Rec 25 and Rec 27 proteins build the axis structure near the binding sites of cohesin Rec8 (44,55,58,59). If recombination processes geometrically occur in the axis after DSB formation, it is plausible that the contact of axis proteins to the DSB hotspot site is beneficial for the coupling of DSB formation and the subsequent recombination processes at the same location. The HORMA domain of Hop1 is also found in the spindle assembly checkpoint protein Mad2 in centromeres and is known to enable the dynamic dissociation of centromeres depending on spindle checkpoint (60). Hop1 may have a dynamic and regulatory function and be responsible for controlling the amount of DSB depending on the cell cycle, checkpoint and chromosome domains, compared to the more static function of Rec10.

### Evolutionary conservation of axial components, the DSB forming complex and their interactions

The results of this study suggest the presence of a universal mechanism for the interaction between axis proteins and the DSB forming complex in eukaryotes to activate meiotic DSB formation. Recent analysis has revealed that most of the factors related to meiotic DSB formation are evolutionarily conserved. For instance, Hop1 is conserved among budding and fission yeasts (Hop1), mammals (HORMAD proteins) and a recent study revealed sequence homology among fission yeast Rec15, budding yeast Mer2 and mammalian IHO1 (53) (see Table 2). In particular, mammalian IHO1 has been identified as a specific binder of HORMAD1 (30), though the interaction between *S. cerevisiae* Hop1 and Mer2 has yet to be demonstrated.

The budding yeast homolog of Rec10 is Red1, and their possible mammalian counterpart may be a synaptonemal and axial protein, SYCP2. Importantly, the N-terminal domains of these Rec10-related proteins are predicted to form a common secondary structure (see Supplementary Figure S7F). In budding yeast, the interaction between Red1 and Hop1 has been reported (41,61), and an analysis of mouse previously has demonstrated that the HORMAD1 protein co-immunoprecipitates with SYCP 2/3 (62). Furthermore, recent studies reported interaction between meiotic HORMAD proteins and Red1/SYCP2/ASY3 in budding yeast, mammals and plants (63,64), implying conserved architecture of axial proteins. Those are consistent with our results that fission yeast Hop1 interacts with Rec10.

It is likely that the domains involved in these intermolecular interactions are at least structurally conserved. For example, the C-terminal domain of Rec15, which is responsible for the interaction with Hop1, is conserved in various taxa including fungi, mammals and plants (53). Prediction of secondary structure suggests that the interface of Rec10 for Rec15 binding is also preserved in fungi and mammals (see Supplementary Figure S7F). The domain of Rec10 for its interaction with Hop1 is also located at close proximity to the conserved N-terminal domain. The proximity of the two interaction domains of Rec10 for Rec15 and Hop1 suggests synergistic mechanisms for DSB regulation by Rec15 and Hop1.

Since fission yeast does not form an authentic SC structure, the functions of *S. pombe* Hop1 are likely to be simplified to their roles in DSB formation (38). However, such conserved domains of Hop1/HORMAD1 proteins in budding yeast and mammals have more diverse roles in meiotic recombination, such as SC formation (17,37,65) and DSB homeostasis (66,67). The Hop1/HORMAD1 may couples DSB formation with the cell cycle, meiotic DSB checkpoint and chromosomal domain structure though its regulatory mechanism yet to be known. It would be intriguing future experiments to introduce point mutations in the conserved domains of mammalian and budding yeast Hop1 to evaluate the influence on meiotic DSB formation and recombination.

## DATA AVAILABILITY

The ChIP-sequencing data is available at DDBJ (http://www.ddbj.nig.ac.jp) under accession number DRA007562.

## Supplementary Material

gkz754_Supplemental_Files
