# Supplementary material for: Conserved HORMA domain-containing protein Hop1 stabilizes interaction between proteins of meiotic DNA break hotspots and chromosome axis
Source: Nucleic Acids Res. 2019 Sep 6;47(19):10166–80. doi: 10.1093/nar/gkz754 (PMC6821256; doi:10.1093/nar/gkz754)
Supplement: gkz754_Supplemental_Files [file gkz754_supplemental_files.zip › 190711Supplementary Table.docx]

Supplementary Table 1

Plasmids used in this paper

| #1 | pGADT7-Hop1 (Full Length) | *1 |
| --- | --- | --- |
| #2 | pGADT7-Hop1 N (1-300a.a.) | *1 |
| #3 | pGADT7-Hop1 C (301-528a.a.) | *1 |
| #4 | pGADT7-Hop1 (9A-mutant) | Mutagenesis from #1 |
| #5 | pGADT7-Rec15 (Full Length) | *1 |
| #6 | pGADT7-Rec15 ∆CT (1-160a.a.) | Mutagenesis from #5 |
| #7 | pGADT7-Rec25 (Full Length) | *2 |
| #8 | pGBKT7-Hop1 | *1 |
| #9 | pGBKT7-Rec15 | *2 |
| #10 | pGBKT7-Mde2 | *2 |
| #11 | pGBKT7-Rec10 (Full Length) | *2 |
| #12 | pGBKT7-Rec10 (E309A) | Mutagenesis from #11 |
| #13 | pGBKT7-Rec10 (P348G K349G) | Mutagenesis from #11 |
| #14 | pGBKT7-Rec10 (291-791a.a.) | Mutagenesis from #11 |
| #15 | pGBKT7-Rec10 (449-791a.a.) | Mutagenesis from #11 |
| #16 | pGBKT7-Rec10 (1-651a.a.) | Mutagenesis from #11 |
| #17 | pGBKT7-Rec10 (1-496a.a.) | Mutagenesis from #11 |
| #18 | pGBKT7-Rec10 (1-348a.a.) | Mutagenesis from #11 |
| #19 | pGBKT7-Rec10 (1-193a.a.) | Mutagenesis from #11 |
| #20 | pAde1 | *2 |
| #21 | pAde1-Hop1 | Hop1 insertion to #20 |
| #22 | pAde1-Hop1 (9A-mutant) | Hop1 insertion to #20 |

*1 Plasmid used in Sakuno and Watanabe 2015

*2 Plasmid used in Miyoshi et.al. 2012

Supplementary Table 2

Strains used in this study

| Figure | Strain | Genotype | Soruce |
| --- | --- | --- | --- |
| 1A-C | YZ980 | *h^-^ pat1-114 hop1^+^-GFP:kanMX6* | *1 |
|  | OTM416 | *h^-^ pat1-114 ade6-M26 ura4-D18 rec15^+^-3FLAG:kanMX6 rec7^+^-3HA:ura4^+^* | *2 |
|  | OTM595 | *h^-^ pat1-114 ade6-M26 ura4-D18 rec10^+^-3FLAG:kanMX6 rec7^+^-3HA:ura4^+^* | *2 |
|  | KR306 | *h^-^ pat1-114 ade6-M26 ura4-D18 hop1:: ura4^+^ Z::P_hop1_-hop1^+^-3FLAG-T_hop1_-nat^r^* | *1 |
| 1D | KR98 | *h^-^ pat1-114 ade6-M26* | *1 |
|  | KR306 | *h^-^ pat1-114 ade6-M26 ura4-D18 hop1:: ura4^+^ Z::P_hop1_-hop1^+^-3FLAG-T_hop1_-nat^r^* | *1 |
|  | KR308 | *h^-^ pat1-114 ade6-M26 ura4-D18 rec10::hyg^r^ hop1:: ura4^+^ Z::P_hop1_-hop1^+^-3FLAG-T_hop1_-nat^r^* | *1 |
|  | KR310 | *h^-^ pat1-114 ade6-M26 ura4-D18 rec15::hyg^r^ hop1:: ura4^+^ Z::P_hop1_-hop1^+^-3FLAG-T_hop1_-nat^r^* | *1 |
| 1E | KR306 | *h^-^ pat1-114 ade6-M26 ura4-D18 hop1:: ura4^+^ Z::P_hop1_-hop1^+^-3FLAG-T_hop1_-nat^r^* | *1 |
|  | KR308 | *h^-^ pat1-114 ade6-M26 ura4-D18 rec10::hyg^r^ hop1:: ura4^+^ Z::P_hop1_-hop1^+^-3FLAG-T_hop1_-nat^r^* | *1 |
|  | KR310 | *h^-^ pat1-114 ade6-M26 ura4-D18 rec15::hyg^r^ hop1:: ura4^+^ Z::P_hop1_-hop1^+^-3FLAG-T_hop1_-nat^r^* | *1 |
| 2C | KR224 | *h^-^ pat1-114 ade6-M26 ura4-D18 hop1:: ura4^+^ Z::P_hop1_-hop1^+^-3HA-T_hop1_-nat^r^* | *1 |
|  | KR229 | *h^-^ pat1-114 ade6-M26 ura4-D18 rec10^+^-3FLAG:kanMX6 hop1:: ura4^+^ Z::P_hop1_-hop1^+^-3HA-T_hop1_-nat^r^* | *1 |
| 2D | KR224 | *h^-^ pat1-114 ade6-M26 ura4-D18 hop1:: ura4^+^ Z::P_hop1_-hop1^+^-3HA-T_hop1_-nat^r^* | *1 |
|  | KR228 | *h^-^ pat1-114 ade6-M26 ura4-D18 rec15^+^-3FLAG:kanMX6 hop1:: ura4^+^ Z::P_hop1_-hop1^+^-3HA-T_hop1_-nat^r^* | *1 |
|  | KR231 | *h^-^ pat1-114 ade6-M26 ura4-D18 rec15∆CT(20aa)-3FLAG:kanMX6 hop1∆ura4+ P_hop1_-hop1^+^-HA-T_hop1_- nat^r^* | *1 |
| 3A | OTM557 | *h- pat1-114 ade6-M26 ura4-D18 rec7+-3HA:ura4+* | *2 |
|  | OTM595 | *h^-^ pat1-114 ade6-M26 ura4-D18 rec10^+^-3FLAG:kanMX6 rec7^+^-3HA:ura4^+^* | *2 |
|  | KR39 | *h^-^ pat1-114 ade6-M26 ura4-D18 rec10^+^-3FLAG:kanMX6 rec7^+^-3HA:ura4^+^ hop1::bsd^r^* | *1 |
|  | OTM607 | *h^-^ pat1-114 ade6-M26 ura4-D18 rec15::hyg^r^ rec10^+^-3FLAG:kanMX6 rec7^+^-3HA:ura4^+^* | *2 |
| 3B | OTM595 | *h^-^ pat1-114 ade6-M26 ura4-D18 rec10^+^-3FLAG:kanMX6 rec7^+^-3HA:ura4^+^* | *2 |
|  | KR144 | *h^-^ pat1-114 ade6-M26 ura4-D18 hop1-9A rec10^+^-3FLAG:kanMX6 rec7^+^-3HA:ura4^+^* | *1 |
|  | KR39 | *h^-^ pat1-114 ade6-M26 ura4-D18 rec10^+^-3FLAG:kanMX6 rec7^+^-3HA:ura4^+^ hop1::bsd^r^* | *1 |
|  | OTM607 | *h^-^ pat1-114 ade6-M26 ura4-D18 rec15::hyg^r^ rec10^+^-3FLAG:kanMX6 rec7^+^-3HA:ura4^+^* | *2 |
| 3C,D | OTM595 | *h^-^ pat1-114 ade6-M26 ura4-D18 rec10^+^-3FLAG:kanMX6 rec7^+^-3HA:ura4^+^* | *2 |
|  | KR39 | *h^-^ pat1-114 ade6-M26 ura4-D18 rec10^+^-3FLAG:kanMX6 rec7^+^-3HA:ura4^+^ hop1::bsd^r^* | *1 |
|  | OTM607 | *h^-^ pat1-114 ade6-M26 ura4-D18 rec15::hyg^r^ rec10^+^-3FLAG:kanMX6 rec7^+^-3HA:ura4^+^* | *2 |
| 4A | OTM557 | *h- pat1-114 ade6-M26 ura4-D18 rec7+-3HA:ura4+* | *2 |
|  | OTM416 | *h^-^ pat1-114 ade6-M26 ura4-D18 rec15^+^-3FLAG:kanMX6 rec7^+^-3HA:ura4^+^* | *2 |
|  | KR38 | *h^-^ pat1-114 ade6-M26 ura4-D18 hop1::bsd^r^ rec15^+^-3FLAG:kanMX6 rec7^+^-3HA:ura4^+^* | *1 |
|  | OTM578 | *h^-^ pat1-114 ade6-M26 ura4-D18 rec10::hyg^r^ rec15^+^-3FLAG:kanMX6 rec7^+^-3HA:ura4^+^* | *2 |
|  | KR95 | *h^-^ pat1-114 ade6-M26 ura4-D18 rec15∆CT(20aa)-3FLAG:kanMX6 rec7^+^-3HA:ura4^+^* | *1 |
| 4B | OTM416 | *h^-^ pat1-114 ade6-M26 ura4-D18 rec15^+^-3FLAG:kanMX6 rec7^+^-3HA:ura4^+^* | *2 |
|  | KR143 | *h^-^ pat1-114 ade6-M26 ura4-D18 hop1-9A rec15^+^-3FLAG:kanMX6 rec7^+^-3HA:ura4^+^* | *1 |
|  | KR38 | *h^-^ pat1-114 ade6-M26 ura4-D18 hop1::bsd^r^ rec15^+^-3FLAG:kanMX6 rec7^+^-3HA:ura4^+^* | *1 |
|  | OTM578 | *h^-^ pat1-114 ade6-M26 ura4-D18 rec10::hyg^r^ rec15^+^-3FLAG:kanMX6 rec7^+^-3HA:ura4^+^* | *2 |
|  | KR95 | *h^-^ pat1-114 ade6-M26 ura4-D18 rec15∆CT(20aa)-3FLAG:kanMX6 rec7^+^-3HA:ura4^+^* | *1 |
| 4C,D | OTM416 | *h^-^ pat1-114 ade6-M26 ura4-D18 rec15^+^-3FLAG:kanMX6 rec7^+^-3HA:ura4^+^* | *2 |
|  | KR38 | *h^-^ pat1-114 ade6-M26 ura4-D18 hop1::bsd^r^ rec15^+^-3FLAG:kanMX6 rec7^+^-3HA:ura4^+^* | *1 |
|  | OTM578 | *h^-^ pat1-114 ade6-M26 ura4-D18 rec10::hyg^r^ rec15^+^-3FLAG:kanMX6 rec7^+^-3HA:ura4^+^* |  |
| 5C | KR261 | *h^-^ ade6-469 leu1-32* | *1 |
|  | KR262 | *h^+^ ade6-M26 his5-303* | *1 |
|  | KR267 | *h^-^ ade6-469 leu1-32 rec10(P348G K349G)* | *1 |
|  | KR268 | *h^+^ ade6-M26 his5-303 rec10(P348G K349G)* | *1 |
|  | KR300 | *h^-^ ade6-469 leu1-32 rec10(E309A)* | *1 |
|  | KR301 | *h^+^ ade6-M26 his5-303 rec10(E309A)* | *1 |
|  | KR25 | *h^-^ ade6-469 ura4-D18 leu1-32 hop1:: ura4^+^* | *1 |
|  | KR28 | *h^+^ ade6-M26 ura4-D18 his5-303 hop1:: ura4^+^* | *1 |
|  | KR238 | *h^-^ ade6-469 ura4-D18 leu1-32 rec10(P348G K349G) hop1:: ura4^+^* | *1 |
|  | KR239 | *h^+^ ade6-M26 ura4-D18 his5-303 rec10(P348G K349G) hop1:: ura4^+^* | *1 |
|  | KR302 | *h^-^ ade6-469 ura4-D18 leu1-32 rec10(E309A) hop1:: ura4^+^* | *1 |
|  | KR303 | *h^+^ ade6-M26 ura4-D18 his5-303 rec10(E309A) hop1:: ura4^+^* | *1 |
|  | KR42 | *h^-^ ade6-469 ura4-D18 leu1-32 rec10:: ura4^+^* | *1 |
|  | KR43 | *h^+^ ade6-M26 ura4-D18 his5-303 rec10:: ura4^+^* | *1 |
|  | KR58 | *h^-^ ade6-469 ura4-D18 leu1-32 rec15:: ura4^+^* | *1 |
|  | KR59 | *h^+^ ade6-M26 ura4-D18 his5-303 rec15:: ura4^+^* | *1 |
|  | KR413 | *h^-^ ade6-469 leu1-32 rec15(∆CT20aa)* | *1 |
|  | KR414 | *h^+^ ade6-M26 his5-303 rec15(∆CT20aa)* | *1 |
| S1A | YZ980 | *h^-^ pat1-114 hop1^+^-GFP:kanMX6* | *1 |
| S1B | KR306 | *h^-^ pat1-114 ade6-M26 ura4-D18 hop1:: ura4^+^ Z::P_hop1_-hop1^+^-3FLAG-T_hop1_-nat^r^* | *1 |
| S1C | OTM416 | *h^-^ pat1-114 ade6-M26 ura4-D18 rec15^+^-3FLAG:kanMX6 rec7^+^-3HA:ura4^+^* | *2 |
| S1D | KR38 | *h^-^ pat1-114 ade6-M26 ura4-D18 hop1::bsd^r^ rec15^+^-3FLAG:kanMX6 rec7^+^-3HA:ura4^+^* | *1 |
| S1E | OTM578 | *h^-^ pat1-114 ade6-M26 ura4-D18 rec10::hyg^r^ rec15^+^-3FLAG:kanMX6 rec7^+^-3HA:ura4^+^* | *2 |
| S1F | OTM595 | *h^-^ pat1-114 ade6-M26 ura4-D18 rec10^+^-3FLAG:kanMX6 rec7^+^-3HA:ura4^+^* | *2 |
| S1G | KR39 | *h^-^ pat1-114 ade6-M26 ura4-D18 rec10^+^-3FLAG:kanMX6 rec7^+^-3HA:ura4^+^ hop1::bsd^r^* | *1 |
| S1H | OTM607 | *h^-^ pat1-114 ade6-M26 ura4-D18 rec15::hyg^r^ rec10^+^-3FLAG:kanMX6 rec7^+^-3HA:ura4^+^* | *2 |
| S2 | YZ980 | *h^-^ pat1-114 hop1^+^-GFP:kanMX6* | *1 |
|  | KR306 | *h^-^ pat1-114 ade6-M26 ura4-D18 hop1:: ura4^+^ Z::P_hop1_-hop1^+^-3FLAG-T_hop1_-nat^r^* | *1 |
|  | OTM416 | *h^-^ pat1-114 ade6-M26 ura4-D18 rec15^+^-3FLAG:kanMX6 rec7^+^-3HA:ura4^+^* | *2 |
|  | OTM595 | *h^-^ pat1-114 ade6-M26 ura4-D18 rec10^+^-3FLAG:kanMX6 rec7^+^-3HA:ura4^+^* | *2 |
| S4C | KR98 | *h^-^ pat1-114 ade6-M26* | *1 |
|  | KR306 | *h^-^ pat1-114 ade6-M26 ura4-D18 hop1:: ura4^+^ Z::P_hop1_-hop1^+^-3FLAG-T_hop1_-nat^r^* | *1 |
|  | KR307 | *h^-^ pat1-114 ade6-M26 ura4-D18 hop1:: ura4^+^ Z::P_hop1_-hop1-9A-3FLAG-T_hop1_-nat^r^* | *1 |
| S4D | KR306 | *h^-^ pat1-114 ade6-M26 ura4-D18 hop1:: ura4^+^ Z::P_hop1_-hop1^+^-3FLAG-T_hop1_-nat^r^* | *1 |
|  | KR307 | *h^-^ pat1-114 ade6-M26 ura4-D18 hop1:: ura4^+^ Z::P_hop1_-hop1-9A-3FLAG-T_hop1_-nat^r^* | *1 |
| S5A | YZ980 | *h^-^ pat1-114 hop1^+^-GFP:kanMX6* | *1 |
|  | OTM595 | *h^-^ pat1-114 ade6-M26 ura4-D18 rec10^+^-3FLAG:kanMX6 rec7^+^-3HA:ura4^+^* | *2 |
|  | KR39 | *h^-^ pat1-114 ade6-M26 ura4-D18 rec10^+^-3FLAG:kanMX6 rec7^+^-3HA:ura4^+^ hop1::bsd^r^* | *1 |
|  | OTM607 | *h^-^ pat1-114 ade6-M26 ura4-D18 rec15::hyg^r^ rec10^+^-3FLAG:kanMX6 rec7^+^-3HA:ura4^+^* | *2 |
| S5B | YZ980 | *h^-^ pat1-114 hop1^+^-GFP:kanMX6* | *1 |
|  | OTM416 | *h^-^ pat1-114 ade6-M26 ura4-D18 rec15^+^-3FLAG:kanMX6 rec7^+^-3HA:ura4^+^* | *2 |
|  | KR38 | *h^-^ pat1-114 ade6-M26 ura4-D18 hop1::bsd^r^ rec15^+^-3FLAG:kanMX6 rec7^+^-3HA:ura4^+^* | *1 |
|  | OTM578 | *h^-^ pat1-114 ade6-M26 ura4-D18 rec10::hyg^r^ rec15^+^-3FLAG:kanMX6 rec7^+^-3HA:ura4^+^* | *2 |
| S6A-D | KR98 | *h^-^ pat1-114 ade6-M26* | *1 |
|  | KR100 | *h- pat1-114 ade6-M26 ura4-D18 hop1:: ura4^+^* | *1 |
|  | KR155 | *h- pat1-114 ade6-M26 hop1-9A* | *1 |
| S6E,F | KR261 | *h^-^ ade6-469 leu1-32* | *1 |
|  | KR262 | *h^+^ ade6-M26 his5-303* | *1 |
|  | KR265 | *h- ade6-469 leu1-32 hop1-9A* | *1 |
|  | KR266 | *h^+^ ade6-M26 his5-303 hop1-9A* | *1 |
|  | KR25 | *h^-^ ade6-469 ura4-D18 leu1-32 hop1:: ura4^+^* | *1 |
|  | KR28 | *h^+^ ade6-M26 ura4-D18 his5-303 hop1:: ura4^+^* | *1 |
| S7D | KR336 | *h^90^ rec10^+^-GFP: ura4^+^* | *1 |
|  | KR337 | *h^90^ rec10(P348G K349G)-GFP: ura4^+^* | *1 |
|  | KR338 | *h^90^ rec10(E309A) -GFP: ura4^+^* | *1 |
| S7E | KR224 | *h^-^ pat1-114 ade6-M26 ura4-D18 hop1:: ura4^+^ Z::P_hop1_-hop1^+^-3HA-T_hop1_-nat^r^* | *1 |
|  | KR229 | *h^-^ pat1-114 ade6-M26 ura4-D18 rec10^+^-3FLAG:kanMX6 hop1:: ura4^+^ Z::P_hop1_-hop1^+^-3HA-T_hop1_-nat^r^* | *1 |
|  | KR407 | *h^-^ pat1-114 ade6-M26 ura4-D18 rec10(E309A)-3FLAG:kanMX6 hop1:: ura4^+^ Z::P_hop1_-hop1^+^-3FLAG-T_hop1_-nat^r^* | *1 |
|  | KR408 | *h^-^ pat1-114 ade6-M26 ura4-D18 rec10(P438G K349G)-3FLAG:kanMX6 hop1:: ura4^+^ Z::P_hop1_-hop1^+^-3FLAG-T_hop1_-nat^r^* | *1 |
| S7C | KR98 | *h^-^ pat1-114 ade6-M26* | *1 |
|  | KR100 | *h- pat1-114 ade6-M26 ura4-D18 hop1:: ura4^+^* | *1 |
|  | KR343 | *h- pat1-114 ade6-M26 rec10(P348GK348G)* | *1 |
|  | KR345 | *h- pat1-114 ade6-M26 ura4-D18 rec10(P348GK348G) hop1:: ura4+* | *1 |
|  | KR344 | *h- pat1-114 ade6-M26 rec10(E309A)* | *1 |
|  | KR346 | *h- pat1-114 ade6-M26 ura4-D18 rec10(E309A) hop1:: ura4^+^* | *1 |
|  | KR354 | *h^-^ pat1-114 ade6-M26 ura4-D18 rec10:: ura4^+^* | *1 |

*1 This study

*2 (Miyoshi et. al. 2012)

Supplementary Table 3

Primers used for ChIP-qPCR assay

|  | name | Primer | Sequence |
| --- | --- | --- | --- |
| Axis | SPBC3H7.03c | CHIP_SPBC3H7.03c_F | TGCCATGAATTCCTATCGTAG |
|  |  | CHIP_SPBC3H7.03c_R | GTTGCAAGGCTTATGTACATAC |
|  | ura1-down | ChIP_ura1down__newF | TATCGTTATGCATCCTTTGC |
|  |  | ChIP_ura1_down_newR | TTCAGTAGCACCCATAACAC |
|  | sib1-down | ChIP_sib1_down_F | TGCACGATACTTGCTTGAAG |
|  |  | ChIP_sib1_down_R | CATCCATCGTATAATGCATG |
|  | SPCC594.01 | ChIP_SPCC594.01_F | GACATACACTATAGATGCAG |
|  |  | ChIP_SPCC594.01_R | GCTGTTCAATAGGCTTCTTG |
| DSB hotspot | mbs1 | CHIP_mbs1_F | AAACACTGGTCATCTCGGAACCAGC |
|  |  | CHIP_mbs1_R | TCCTTTGCAGTGAATGGTTGGGCTAC |
|  | sat1 | ChIP_sat1_F | TCATCTCATTAAGAGTGTTCGG |
|  |  | ChIP_sat1_R | GATGCAAAGCAAATGCGCAAGT |
|  | ste11 | CHIP_ste11_F | TCATCTTTACCTCTACTTTC |
|  |  | CHIP_ste11_R | ACAAGGTTGAGCTTAAAAGG |
|  | mae2 | ChIP_mae2_F | GTTGTAAAGATTGTTCTCGG |
|  |  | ChIP_mae2_R | CTTTAAATGAATGATCCCCC |
| Cold Spot | sib1 | ChIP_sib1_F | TGGCTCTAGATGTATCAGCG |
|  |  | ChIP_sib1_R | ACGGCTGTCGAATCAGTTAC |
|  | dis2 | ChIP_dis2_F | TTGTTTTATTGAAGCTCCGC |
|  |  | ChIP_dis2_R | ATTGTATGCCATTGAATCCC |
|  | ime4 | ChIP_ime4_F | TACAGTTTGAGGATCAGTGC |
|  |  | ChIP_ime4_R | CCATCATTTTGGACACATTG |

Supplementary Table 4

Software used in this study

|  | Refarence | Version |
| --- | --- | --- |
| Bowtie2 | Langmead and Salzberg, 2012(1) | 2.2.9 |
| MACS2 | Zhang et. al. 2008(2) | 2.1.1 |
| Integrative genomics viewer | Robinson et. al. 2011(3) | 2.3.92 |
| IGVtools | Thorvaldsdóttir et.al.2013(4) | 2.3.68 |
| Bedtools | Quinlan and Hall 2010(5) | 2.17.0 |
| R | R Core Team 2018(6) | 3.5.2 |

References

1. Langmead,B. and Salzberg,S.L. (2012) Fast gapped-read alignment with Bowtie 2. *Nat. Methods*, **9**, 357–359.

2. Zhang,Y., Liu,T., Meyer,C.A., Eeckhoute,J., Johnson,D.S., Bernstein,B.E., Nussbaum,C., Myers,R.M., Brown,M., Li,W., *et al.* (2008) Model-based analysis of ChIP-Seq (MACS). *Genome Biol.*, **9**.

3. Robinson,J.T., Thorvaldsdóttir,H., Winckler,W., Guttman,M., Lander,E.S., Getz,G. and Mesirov,J.P. (2011) Integrative genomics viewer. *Nat. Biotechnol.*, **29**, 24–26.

4. Thorvaldsdóttir,H., Robinson,J.T. and Mesirov,J.P. (2013) Integrative Genomics Viewer (IGV): High-performance genomics data visualization and exploration. *Brief. Bioinform.*, **14**, 178–192.

5. Quinlan,A.R. and Hall,I.M. (2010) BEDTools: A flexible suite of utilities for comparing genomic features. *Bioinformatics*, **26**, 841–842.

6. R Core Team (2018). R: A language and environment for statistical computing.
