# Supplementary material for: Conserved HORMA domain-containing protein Hop1 stabilizes interaction between proteins of meiotic DNA break hotspots and chromosome axis
Source: Nucleic Acids Res. 2019 Sep 6;47(19):10166–80. doi: 10.1093/nar/gkz754 (PMC6821256; doi:10.1093/nar/gkz754)
Supplement: gkz754_Supplemental_Files [file gkz754_supplemental_files.zip › 190813_SupFigs.pdf]

Fig. S1 Kariyazono *et al.*

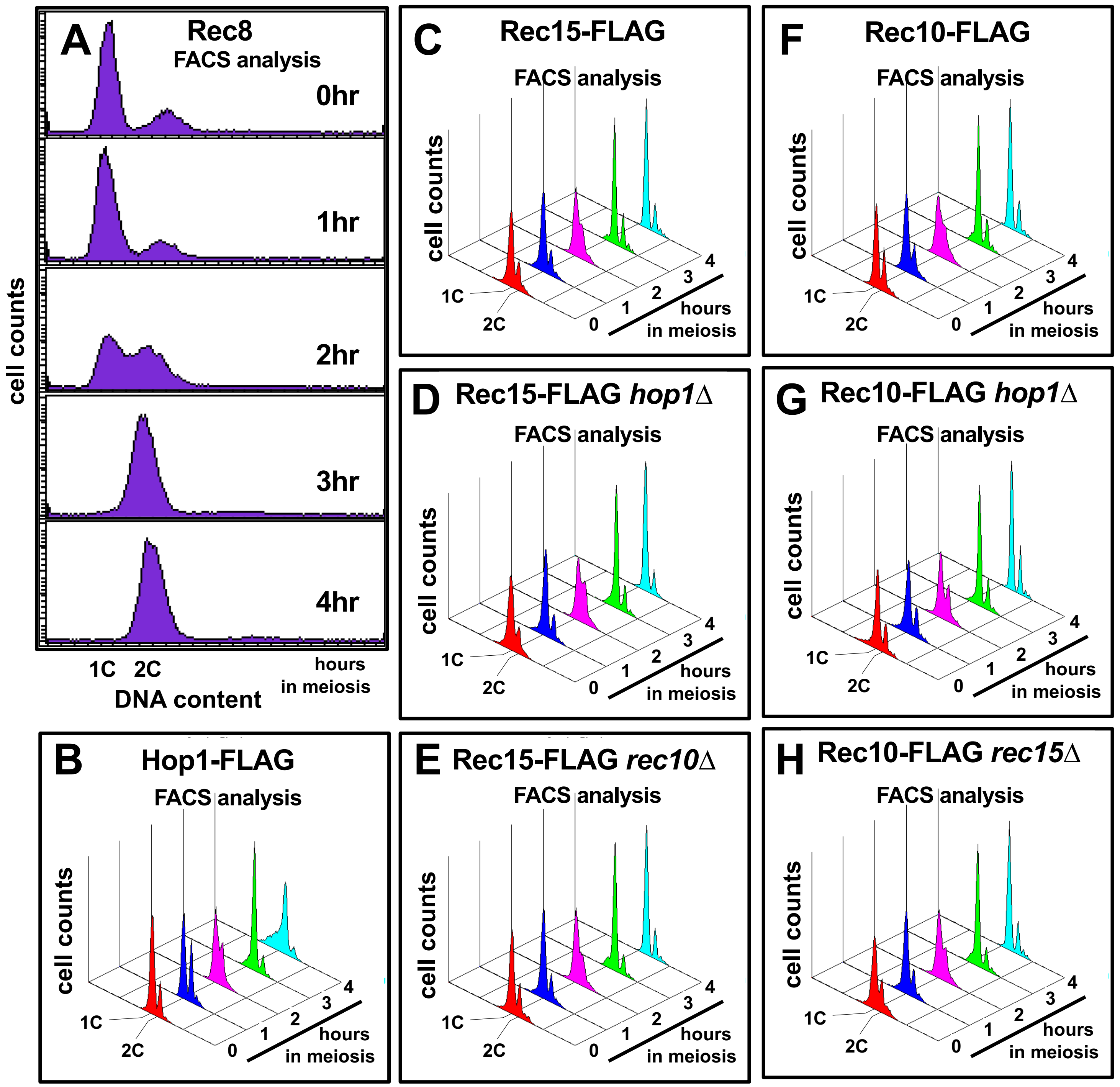

Supplementary Figure S1. Meiotic cell cycle analysis by flow cytometry
(A) Meiotic progression of cells used for Rec8 ChIP experiments. Y- and X-axes represent cell counts and DNA content, respectively. (B)-(H) Meiotic progression of cells expressing FLAG-tagged versions of Hop1, Rec10, and Rec15. Y- and X-axes represent the cell counts and DNA content, respectively. Z-axis indicates the time after meiotic induction.

Fig. S2 Kariyazono *et al.*

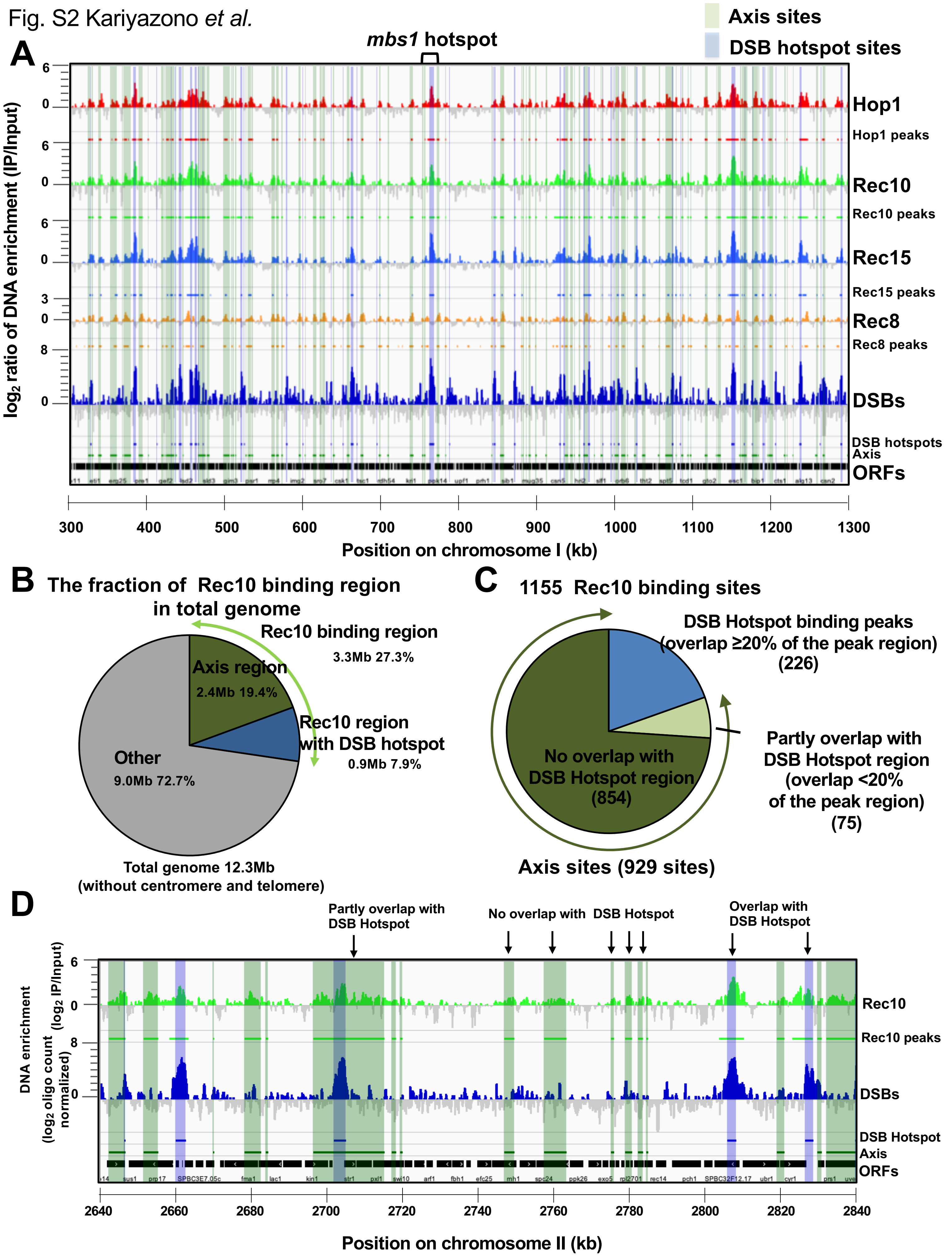

E

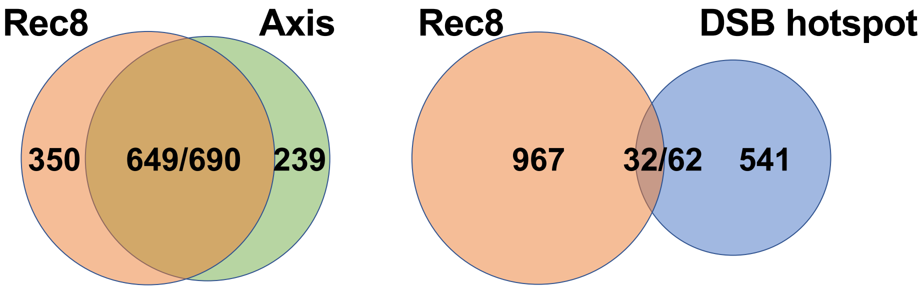
Rec8
Axis
350
649/690
239
Rec8
DSB hotspot
967
32/62
541

F

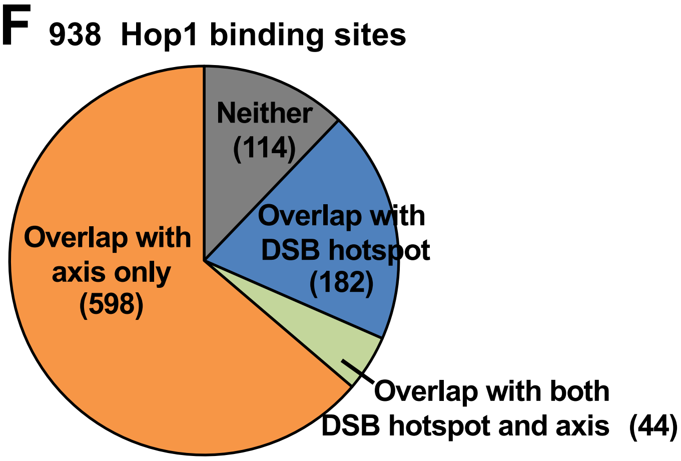
938 Hop1 binding sites
Neither (114)
Overlap with DSB hotspot (182)
Overlap with axis only (598)
Overlap with both DSB hotspot and axis (44)

G

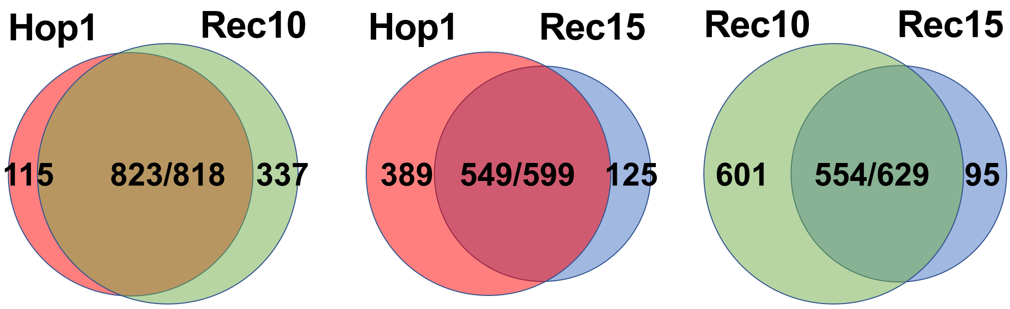
Hop1
Rec10
115
823/818
337
Hop1
Rec15
389
549/599
125
Rec10
Rec15
601
554/629
95

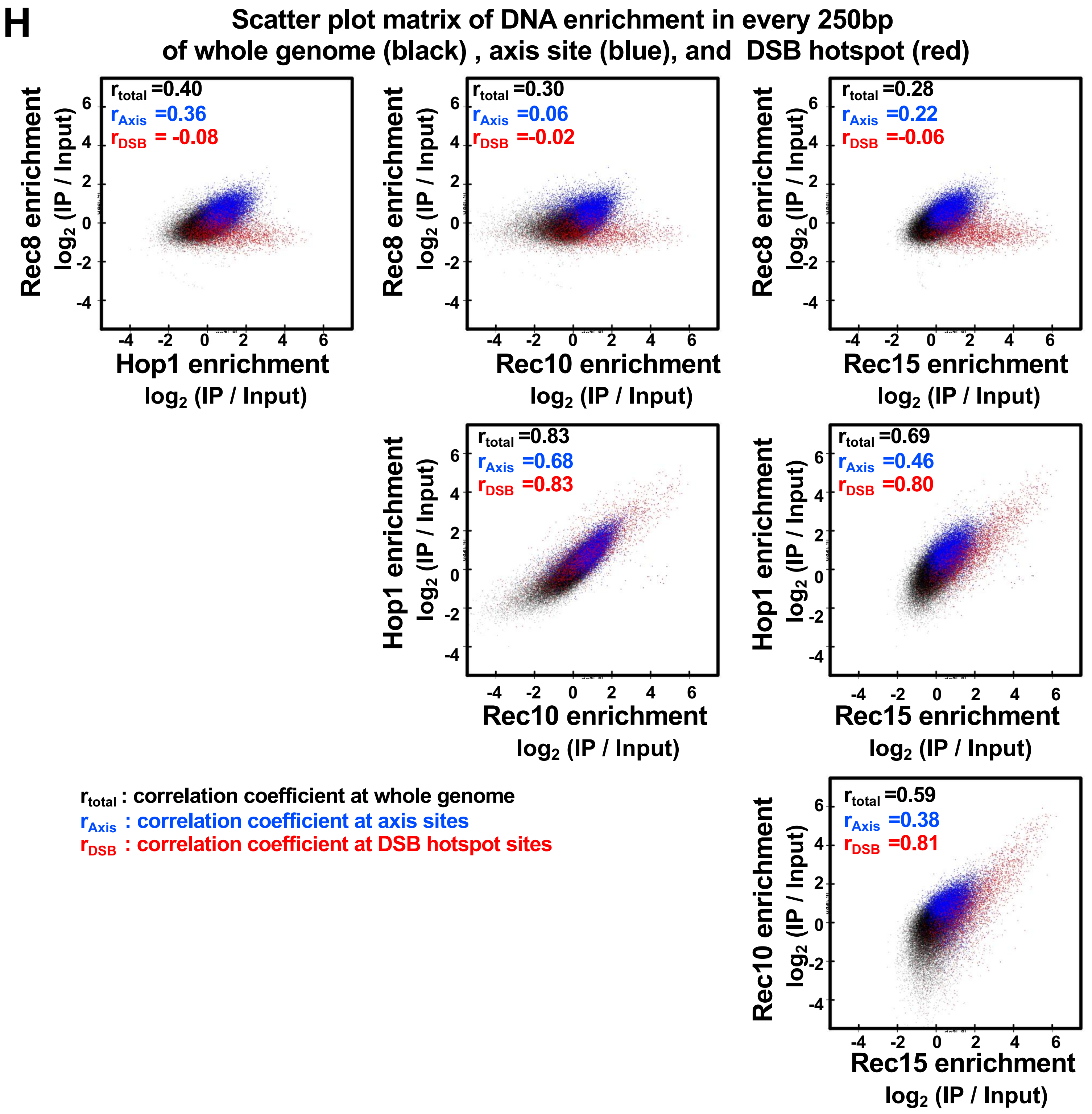

H
Scatter plot matrix of DNA enrichment in every 250bp
of whole genome (black) , axis site (blue), and DSB hotspot (red)
r_total =0.40
r_Axis =0.36
r_DSB = -0.08
Rec8 enrichment
log2 (IP / Input)
Hop1 enrichment
log2 (IP / Input)
r_total =0.30
r_Axis =0.06
r_DSB =-0.02
Rec8 enrichment
log2 (IP / Input)
Rec10 enrichment
log2 (IP / Input)
r_total =0.28
r_Axis =0.22
r_DSB =-0.06
Rec8 enrichment
log2 (IP / Input)
Rec15 enrichment
log2 (IP / Input)
r_total =0.83
r_Axis =0.68
r_DSB =0.83
Hop1 enrichment
log2 (IP / Input)
Rec10 enrichment
log2 (IP / Input)
r_total =0.69
r_Axis =0.46
r_DSB =0.80
Hop1 enrichment
log2 (IP / Input)
Rec15 enrichment
log2 (IP / Input)
r_total : correlation coefficient at whole genome
r_Axis : correlation coefficient at axis sites
r_DSB : correlation coefficient at DSB hotspot sites
r_total =0.59
r_Axis =0.38
r_DSB =0.81
Rec10 enrichment
log2 (IP / Input)
Rec15 enrichment
log2 (IP / Input)

Supplementary Figure S2. ChIP-seq analysis

(A) Wide range views of ChIP-seq data of Rec8, Hop1, Rec10, and Rec15 around a *mbs1* DSB hotspot locus. The Y-axis represents log_2 scaled DNA enrichment (IP-read counts per Input-read counts). Black bars at the bottom indicate open reading frames. Data of DSBs represents the count of Rec12 oligonucleotides described in a previous study.(44).(B) A pie chart showing the fraction of Rec10 binging region in the total genome. (C) A pie chart showing the classification of Rec10 peaks. Rec10 peaks overlapping with DSB hotspot sites with their fraction of greater than 20% are classified as DSB Hotspot binding peaks. Other Rec10 peaks are classified as axis sites.(D) Examples of Rec10 peaks classified into axis sites and DSB overlapping sites.(E) Venn diagrams showing the overlap between Rec8 binding sites and axis sites (left), and between Rec8 binding sites and DSB hotspot sites (right). (F) A pie chart of Hop1 peaks classified by overlapping status with axis sites and/or DSB hotspots. The numbers in parenthesis represent the scale of each category. (G) Venn diagrams showing the overlap between Rec10 sites and Hop1 sites (left), Hop1 sites and Rec15 sites (center), and between Rec10 sites and Rec15 sites (right). The numbers in the diagrams indicate those of each site. (H) Scatter plot matrix of DNA enrichment (log2-scaled IP read counts/Input read counts) for two parameters indicated on the diagonal. Whole genome data points (250 bp each) except for the centromere region and the telomere region were plotted in black dot. Data points within axis sites are indicated in blue dots, and data points within DSB hotspot regions are indicated in red dots. Values of r indicate Pearson correlation coefficients of whole data points (r_{total}), data points within axis sites (r_{Axis}), and data points within DSB hotspot regions (r_{DSB}).

Fig. S3 Kariyazono *et al.*

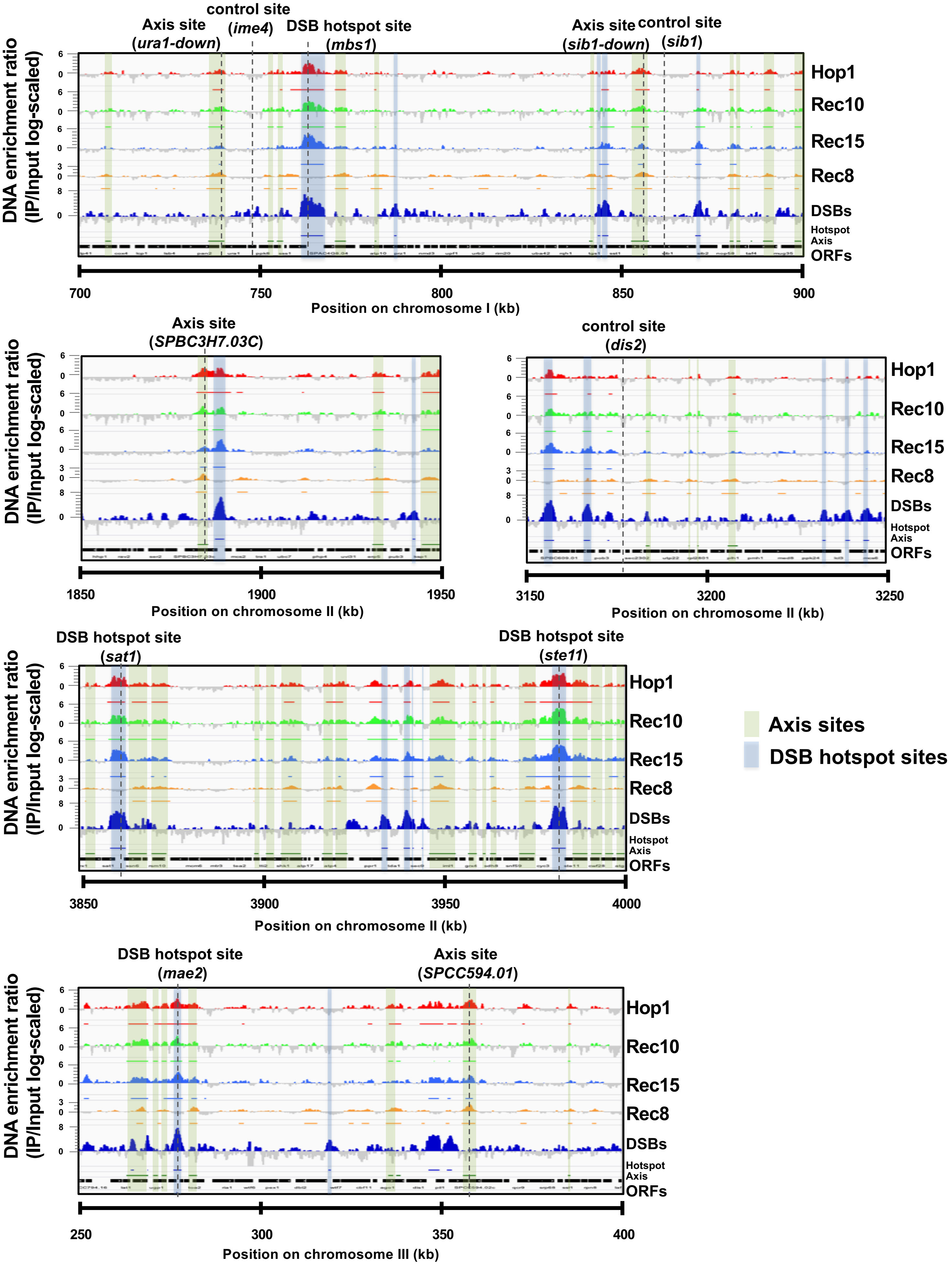

Supplementary Figure S3.

ChIP-seq data of Rec8, Hop1, Rec10, and Rec15, and Rec12 oligo count (DSBs) around Positions of ChIP-qPCR primers tested in this paper (Axis sites; *SPBC3H7.03c, ura1-down, sib1-down, SPCC594.01,* DSB hotspots; *mbs1, sat1, mae2, and ste11*, and control sites; *dis2 ime4,* and *sib1*). The Y-axis represents log2-scaled DNA enrichment (IP read counts/Input read counts). Gray horizontal bars represent open reading flames. Count of Rec12 oligonucleotides are described in previous study. (44) Vertical dotty line indicates precise positions of ChIP-qPCR primers. Bands with blue shading indicates the region of the *mbs1* DSB hotspot and other hotspots. Light green-shaded bands represent the regions of axis sites.

Fig. S4 Kariyazono *et al.*

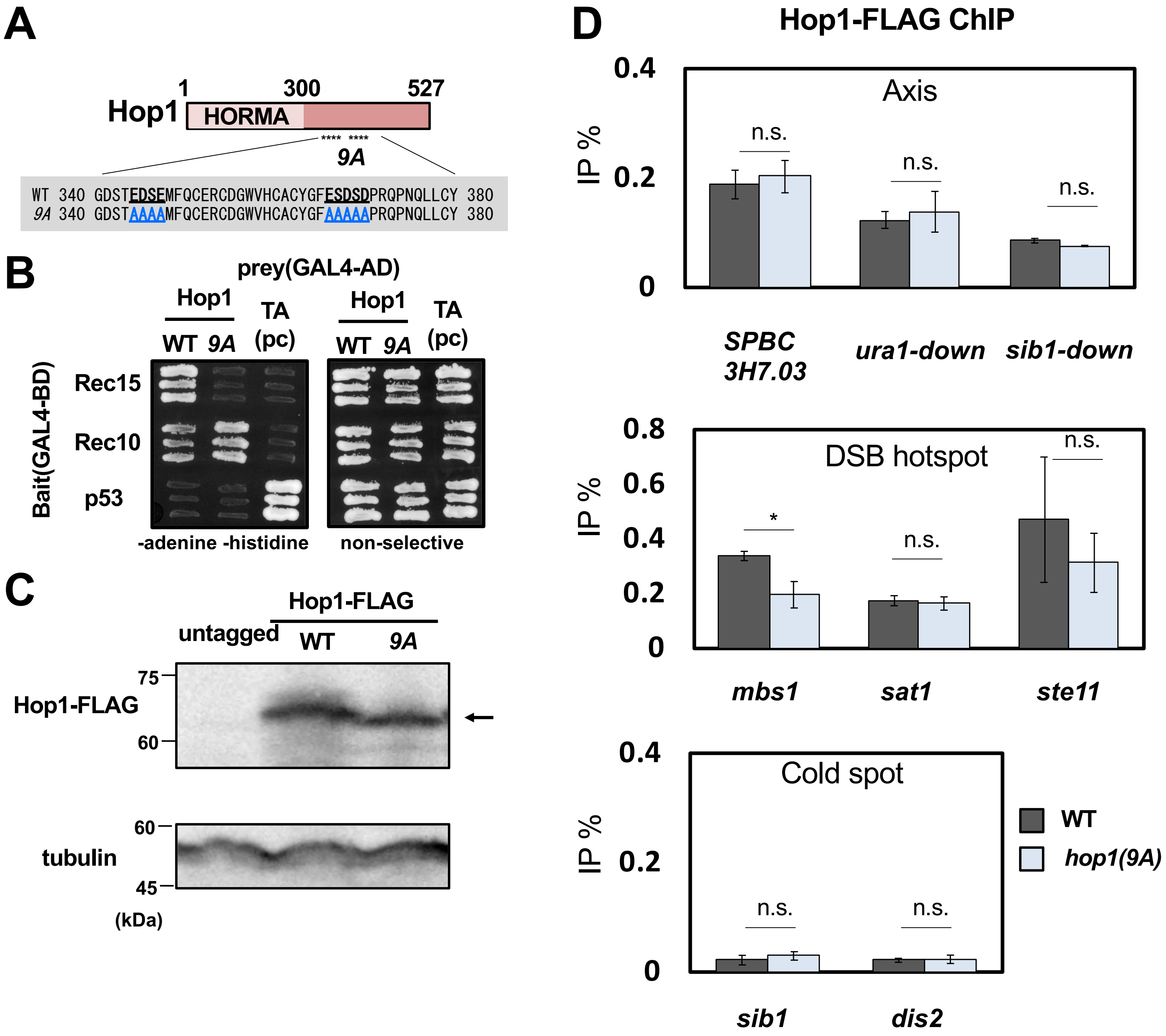

(A) Positions of alanine substitutions in *hop1-9A*. (B) Y2H assay of Hop1 wild type (WT) and *hop1-9A* versus Rec10 and Rec15. (C) Abundance of FLAG-tagged Hop1 and Hop1-9A proteins. (KR306, and KR307, respectively. KR98 was used as untagged control) Cells were harvested at 4 hours after meiotic induction. Proteins were detected by immunoblotting using anti-FLAG antibody. (D) Enrichment of Hop1 and Hop1-9A. ChIP-qPCR assay was performed as in Figure 1D. Error bars represent the S.D. of three biological replicates. n.s indicates non significant (p>5%) and single asterisk(*) indicate significant difference at 5% by two-sided Welch's t test.

Fig. S5 Kariyazono *et al.*

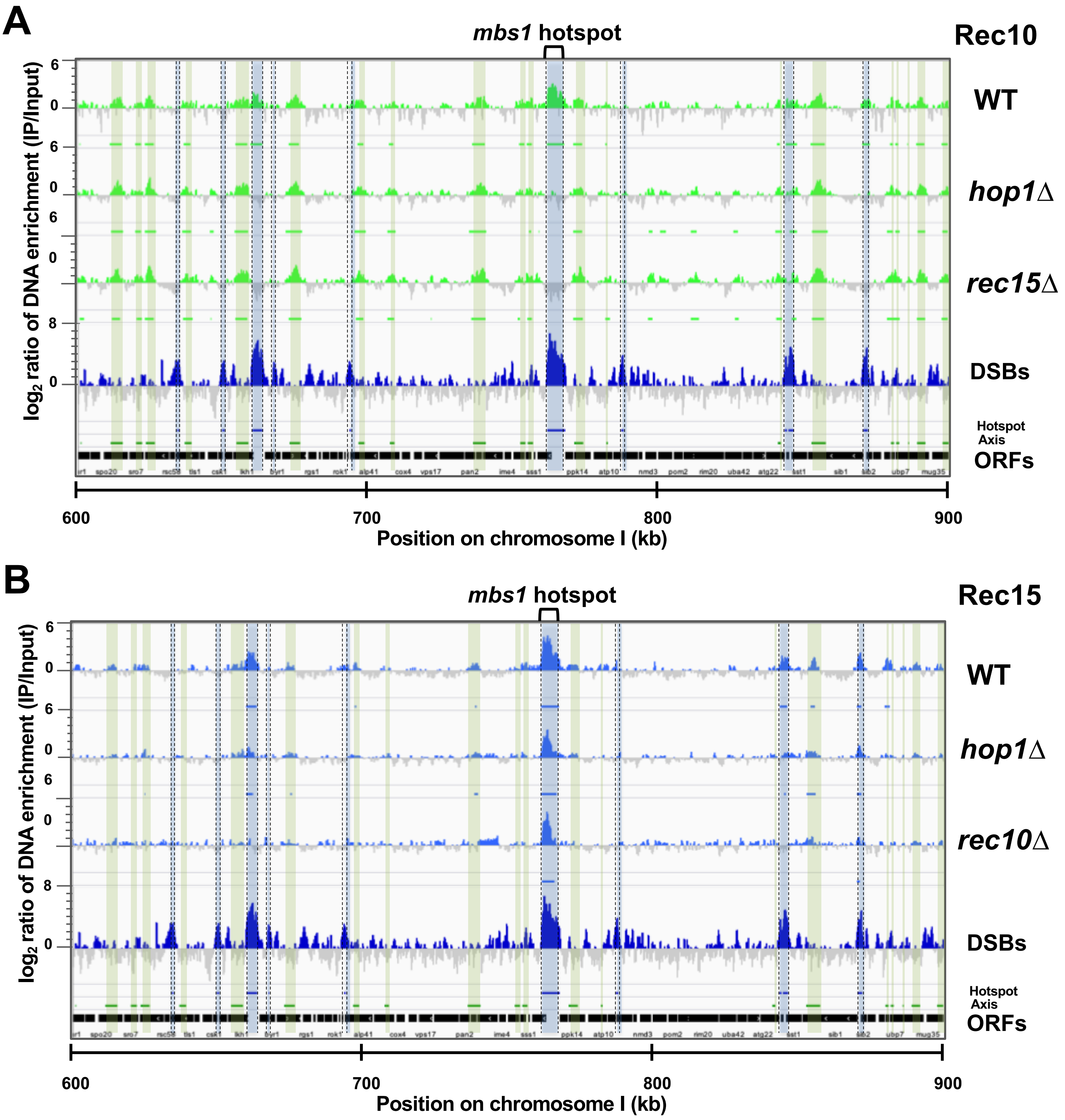

Supplementary Figure S5. Effect of Hop1 deletion on Rec15 and Rec10
(A)ChIP-seq data of Rec10 around *mbs1* DSB hotspot locus in wild type, *hop1*Δ, and *rec15*Δ (OTM595, KR39, and OTM607, respectively). The Y-axis represents log_2-scaled DNA enrichment (IP read counts/Input read counts). Experiments were conducted as in Figure 1A. DSBs represents the count of Rec12 oligonucleotides described in previous study.(44) Gray horizontal bars represent open reading flames. Gray bands between two dotted lines indicate the region of the *mbs1* DSB hotspot. Shaded green boxes represent axis sites. (B) ChIP-seq data of Rec15 around the *mbs1* DSB hotspot in wild type, *hop1*Δ, and *rec10*Δ (OTM416, KR38, and OTM578, respectively). Y-axis and other indications are as described in (A).

Fig. S6 Kariyazono *et al.*

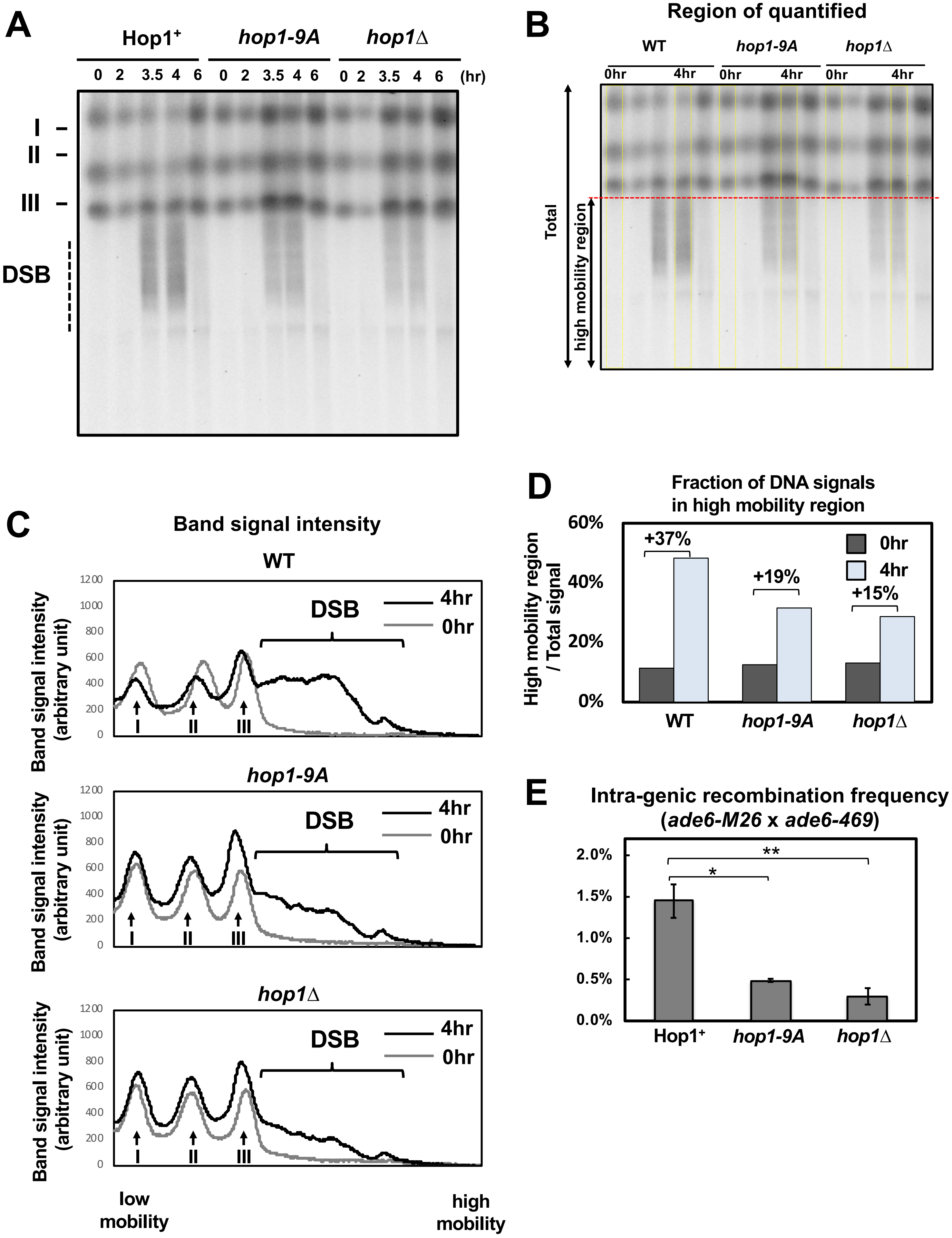

Supplementary Figure S6. Hop1-Rec15 interaction enhances DSB
(A) PFGE images of wild type, *hop1-9A*, and *hop1*Δ (KR98, KR155, and KR100 respectively). The numbers above the images represent the time of cell harvest (0, 2, 3.5, 4, and 6 hours after meiotic induction). The numbers in the left side of the panel indicate the position of *S. pombe* chromosomes (I, II, III). A dashed line represents DSB formation. (B) Region quantified in (C) and (D). Signals in the yellow rectangles were measured as total DNA and signals in regions under the red dotty line were measured as high mobility region in (D).(C) The intensity plots of band signals in each strains in (A) at 0hr and 4hr after meiotic induction (D) Percentage of DNA signals in high mobility region. The quantification of band signals was conducted in each strains in (A) at 0hr and 4hr after meiotic induction. Increments of the percentage are shown in top of bars.(E) Frequency of intergenic recombination ratio of *ade6* were measured in wild type, *hop1-9A*, and *hop1*Δ. Error bars indicate S.D. of three biological replicates. Asterisks * and ** represent a statistical significance at <5% and <1% by two-sided Welch's t test, respectively.

Fig. S7 Kariyazono *et al.*

A

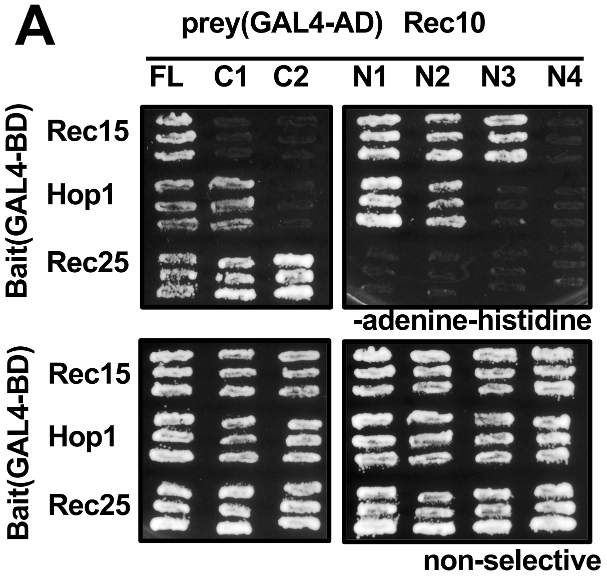

Rec15	Hop1	Rec25	Rec10
+	+	+	FL
-	+	+	C1
-	-	+	C2
+	+	-	N1
+	+	-	N2
+	-	-	N3
-	-	-	N4

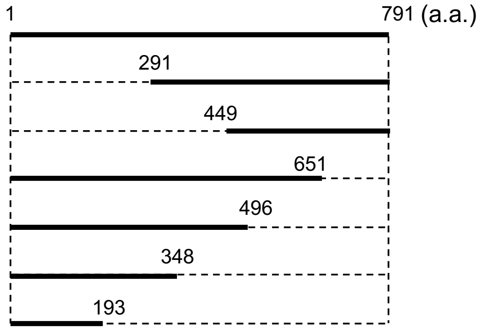

B

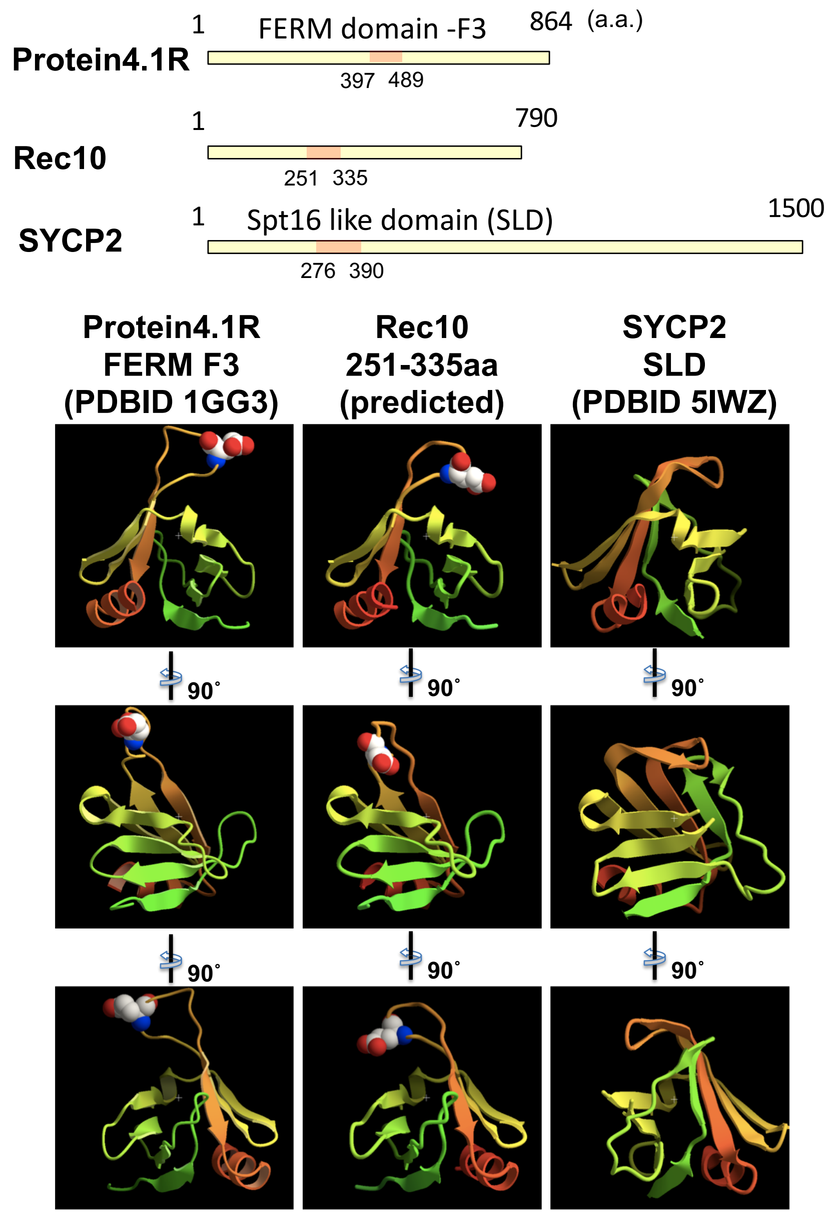

C

Mad2 binding Consensus		R / Kψψ φ P	
Rec10 (Sp)	337	NKLLPAIIVSPKLTLR	353
Red1 (Sc)	336	ETFFHNVNNIPKISEV	352
		:. :: : **::	

ψ ... aliphatic residues (IVLM)
φ ... hydrophobic residues (IVLMF)
K ... budding yeast Red1 K348
PK... fission yeast Rec10 P348K349
* ... identical residues
: ... similar residues
. ... weakly similar residues

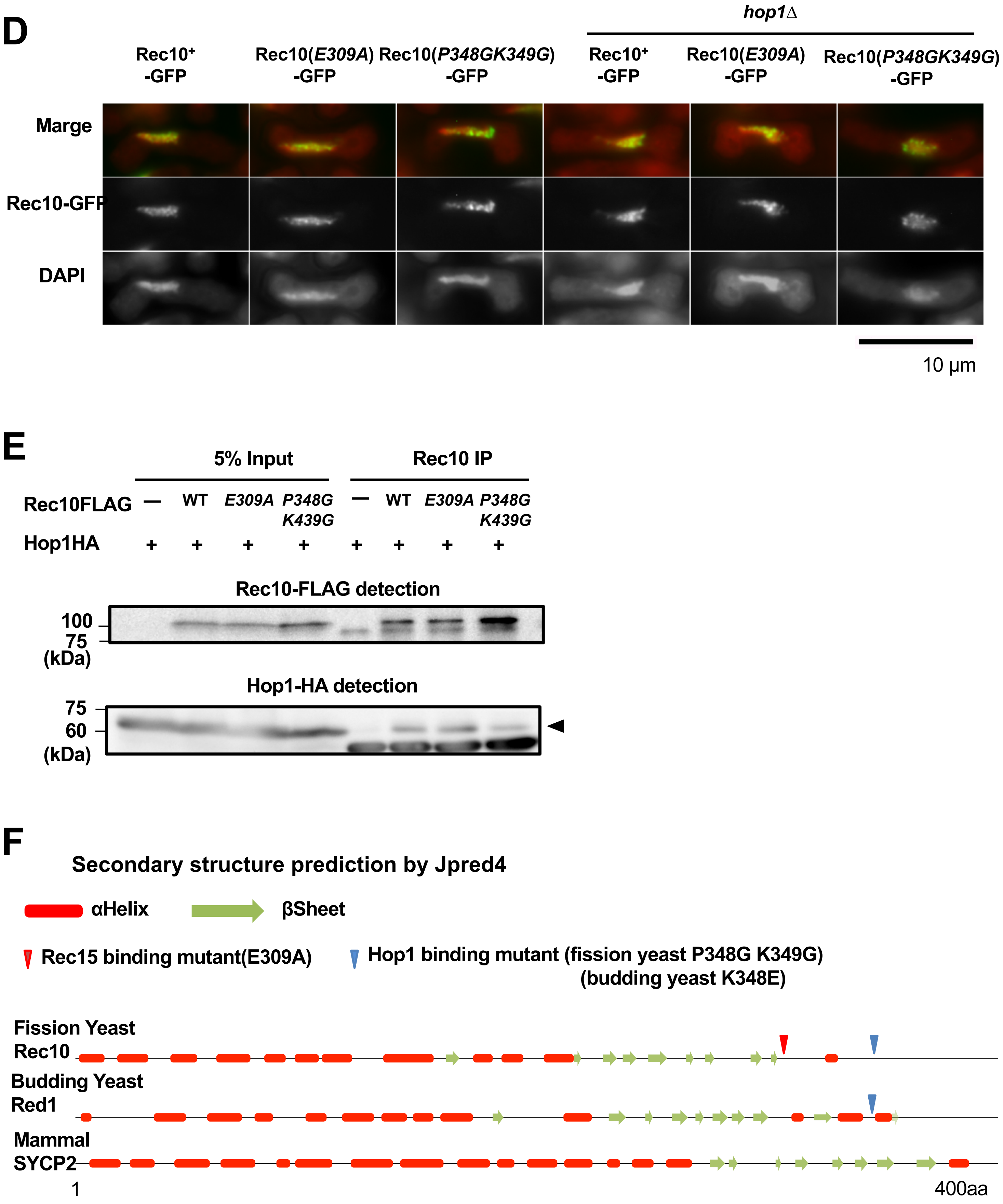

D
hop1Δ
Rec10+ -GFP
Rec10(E309A) -GFP
Rec10(P348GK349G) -GFP
Rec10+ -GFP
Rec10(E309A) -GFP
Rec10(P348GK349G) -GFP
Marge
Rec10-GFP
DAPI
10 μm
E
5% Input
Rec10 IP
Rec10FLAG
—
WT
E309A
P348G K439G
—
WT
E309A
P348G K439G
Hop1HA
+ + + + + + + +
Rec10-FLAG detection
100
75
(kDa)
Hop1-HA detection
75
60
(kDa)
F
Secondary structure prediction by Jpred4
αHelix
βSheet
Rec15 binding mutant(E309A)
Hop1 binding mutant (fission yeast P348G K349G)
(budding yeast K348E)
Fission Yeast Rec10
Budding Yeast Red1
Mammal SYCP2
1
400aa

Supplementary Figure S7. The N-terminus structurally conserved domain of Rec10 is essential for the binding of Rec15 and Rec10

(A) Y2H assay between "baits" (full-length and truncated Rec10) versus *GAL4-AD* "prey" conjugated with Rec15, Hop1, and Rec25. Their interaction capabilities are summarized in the table. (B) (top) The location of structure-predicted region of Rec10 and corresponding domain of Protein 4.1 and SYCP2. (bottom left) A portion of the crystal structure of Protein 4.1. Emphasized residue is glutamate 246, important residue for p55 binding (bottom center). The predicted structure of the N terminus (251-335 amino acids) of fission yeast Rec10. The structure is modelled from Protein 4.1. Emphasized residue is glutamate 309, which was introduced mutation. (bottom right) A portion of the crystal structure of SYCP2 N terminal region. (C) Motif search of HORMA domain binding motif in Rec10 and alignment of Hop1 binding motif of fission yeast Rec10 and budding yeast Red1. Yellow highlight is Mad2 binding consensus motif (R/KψψxφxxxP), where ψ indicates aliphatic residue, φ indicates hydrophobic residues, and x indicates any residues. Red letters indicate mutated residues in fission yeast Rec10. Blue letters indicate important sequence for Hop1 binding in budding yeast Red1. (D) Microscopic observation of GFP-tagged Rec10. Cells were cultured in a sporulation medium, fixed, stained by DAPI, and observed during meiotic prophase.(E) Co-IP experiments for the detection of the interactions of Hop1-HA with Rec10-FLAG, Rec10-E309A, and Rec10-P348G K439G. Co-IP was conducted as in Figure 2C. An arrowhead indicates the position of the Hop1-HA band. An asterisk indicates the position of immunoglobulins. (F) The secondary structure prediction of the N terminus (1-400 amino acids) of fission yeast Rec10, budding yeast Red1, and mammalian SYCP2 were analysed by J pred4. Red and green rectangles represent positions of α-helix and β-sheet, respectively. Red and blue arrowheads indicate positions of E309A and P348G K349G, respectively.

Fig. S8 Kariyazono *et al.*

A

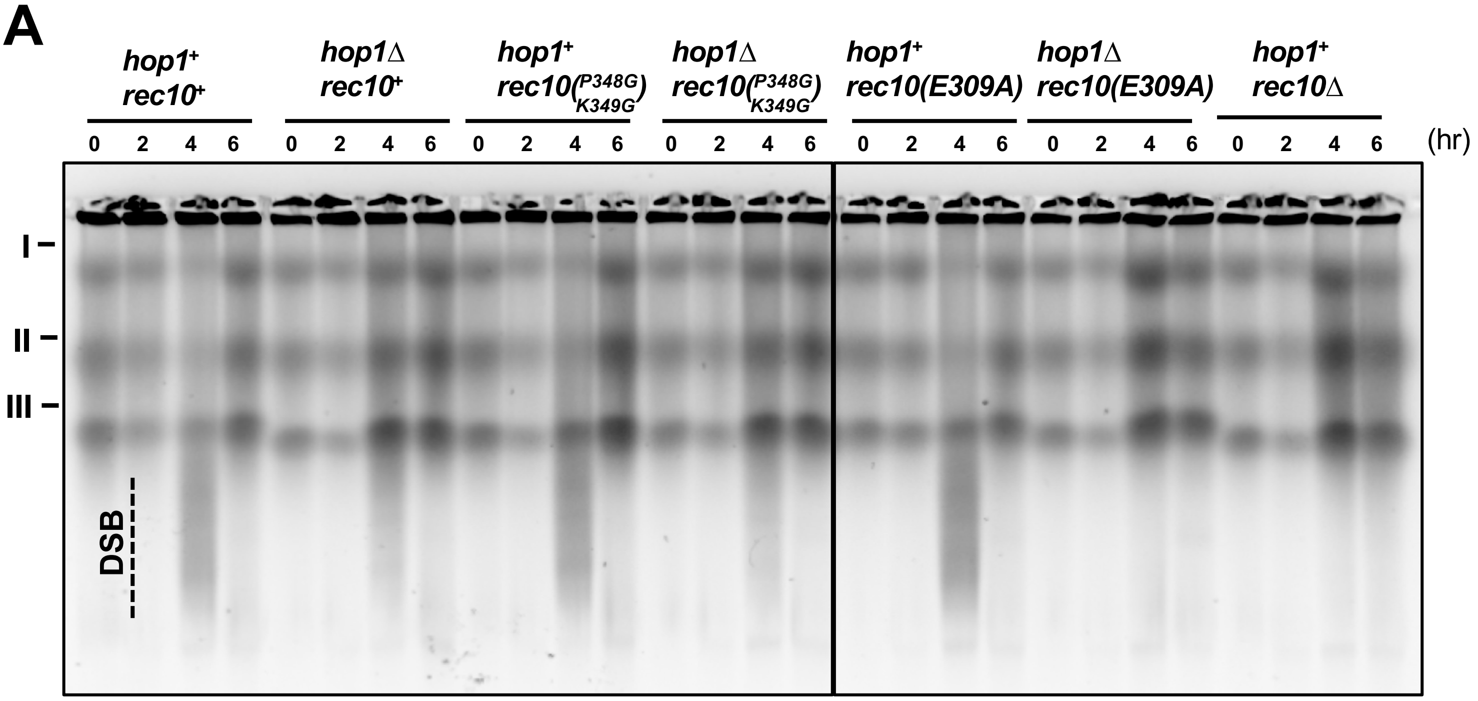

B

Region of quantified

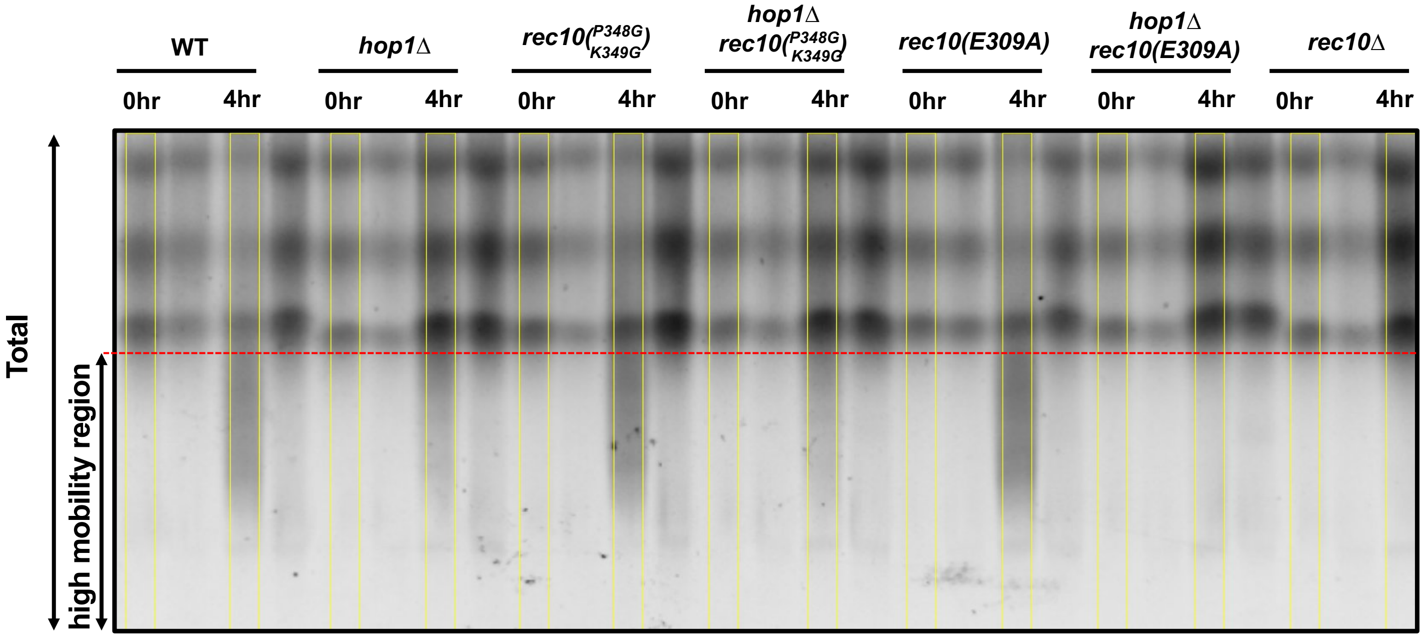

C

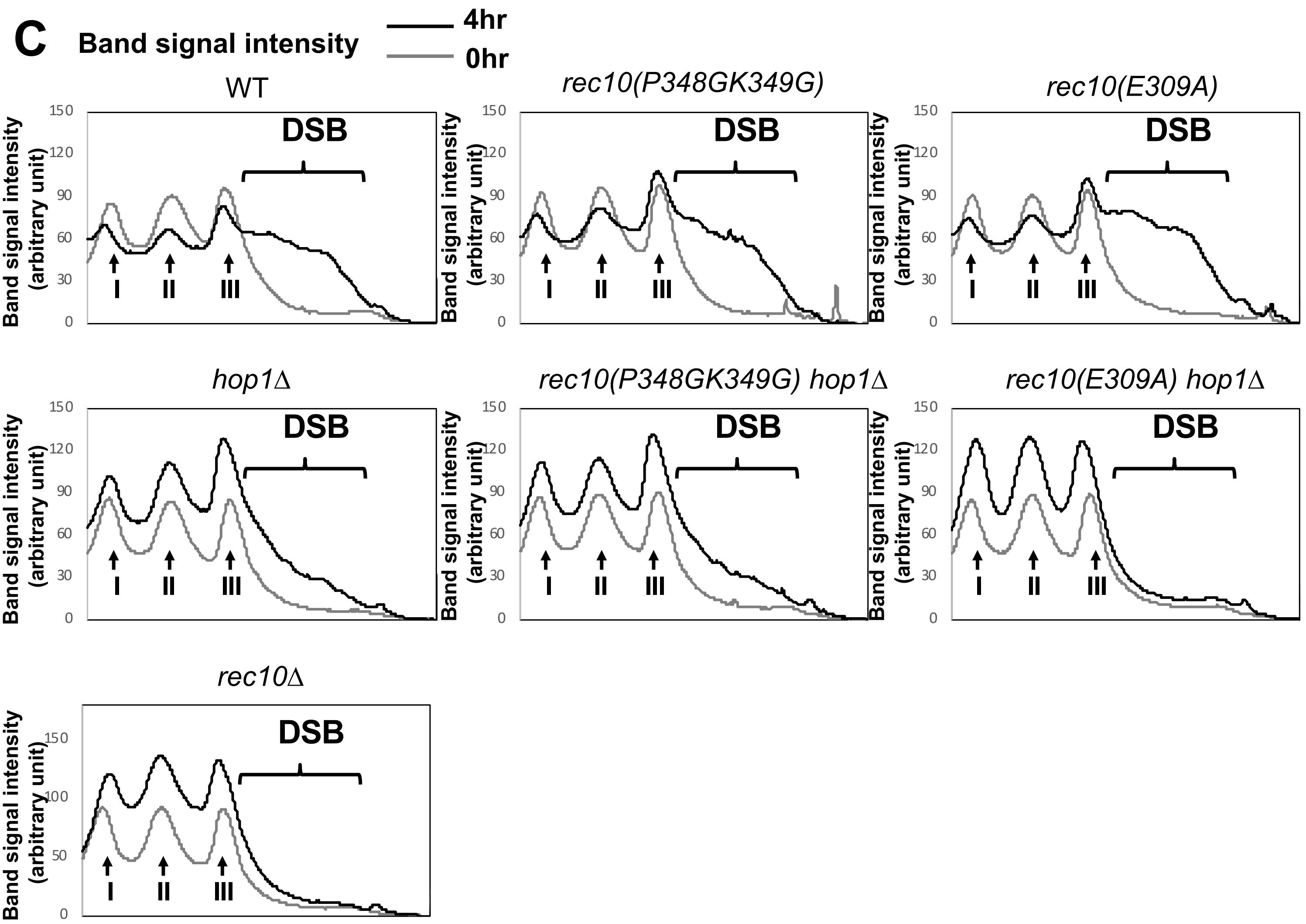
Band signal intensity
4hr
0hr
WT
rec10(P348GK349G)
rec10(E309A)
hop1Δ
rec10(P348GK349G) hop1Δ
rec10(E309A) hop1Δ
rec10Δ
DSB
I
II
III
Band signal intensity (arbitrary unit)
150
120
90
60
30
0
100
50

D

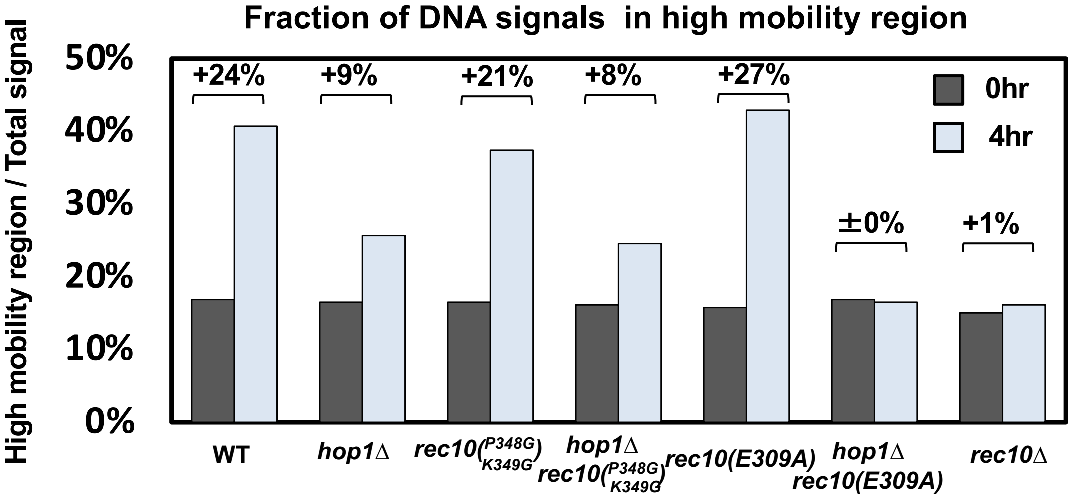
Fraction of DNA signals in high mobility region
High mobility region / Total signal
50%
40%
30%
20%
10%
0%
+24%
+9%
+21%
+8%
+27%
±0%
+1%
0hr
4hr
WT
hop1Δ
rec10(P348G K349G)
hop1Δ rec10(P348G K349G)
rec10(E309A)
hop1Δ rec10(E309A)
rec10Δ

Supplementary Figure S8. (A) PFGE images for the detection of meiotic DSBs in wild type, *hop1Δ*, *rec10P348GK349G*, *hop1Δ-rec10 P348GK349G* double mutant, *rec10E309A*, *hop1Δ-rec10E309A* double mutant, and *rec10Δ* (KR98, KR100, KR343, KR345, KR344, KR346 and KR354 respectively). The numbers above the images represent the time of cell harvest (0, 2, 4 and 6 hours after meiotic induction). The numbers in the left side of the panel indicate the position of *S. pombe* chromosomes (I, II, III). A dashed line represents DSB formation. (B) Region quantified in (C) and (D). Signals in the yellow rectangles were measured as total DNA and signals in regions under the red dotty line were measured as high mobility region in (D). (C) The intensity plots of band signals in each strains in (A) at 0hr and 4hr after meiotic induction. (D) Percentage of DNA signals in high mobility region. The quantification of band signals was conducted in each strains in (A) at 0hr and 4hr after meiotic induction. Increments of the percentage are shown in top of bars
