# Supplementary material for: Conserved HORMA domain-containing protein Hop1 stabilizes interaction between proteins of meiotic DNA break hotspots and chromosome axis
Source: Nucleic Acids Res. 2019 Sep 6;47(19):10166–80. doi: 10.1093/nar/gkz754 (PMC6821256; doi:10.1093/nar/gkz754)
Supplement: gkz754_Supplemental_Files [file gkz754_supplemental_files.zip › supplemental-document.docx]

**Supplementary document**

**Procedure of choosing Rec10 E309A**

Detailed yeast 2 hybrid assay revealed that N-terminus region of Rec10 is responsible for the binding to Rec15 (Supplemental FigureS7A). Although crystal structure of N-terminal domain of SYCP2 (putative mammalian homolog of Rec10) has been determined(1)(PDB ID 5IWZ), SYCP2 has not enough information of binding surface to interactor. To gain Rec10-like structure whose binding surface is well-characterized, the Rec10 N-terminal (201-400) protein structure was predicted using the PHYRE2 Protein Fold Recognition server. The PHYRE2 program revealed that the N terminal structure was similar to a domain called the C-lobe in the erythroid membrane protein 4.1r (PDB ID 1GG3) with 79.9% confidence. Although analogous region is limited, the predicted structure of Rec10 highly resembles actual structure of SYCP2 N terminal domain, indicating the prediction works well. (Supplemental Figure S7B) The C-lobe of erythroid membrane protein 4.1r is known to have a binding surface to the p55 protein (2, 3) (from Y214 to E246). Rec10 E309 (corresponds E246 in protein 4.1R) were substituted to alanine in order to diminish its ability to bind Rec15.

**Procedure of choosing Rec10 P348G K349G**

Previous study on budding yeast reported that Red1 K348 is essential for its binding to Hop1(4). In order to apply this information to fission yeast Rec10, we aligned Rec10 and Red1 based on the previous study (5). As the homology between fission yeast Rec10 and budding yeast Red1 is weak, we also referred to Mad2 binding consensus “R/KψψXφxxP” (6). Among Rec10 residues, we chose P348 from the comparison to Mad2 binding consensus and K349 from the homology to budding yeast Red1 K348 (Supplemental Figure S7C).

1. Feng,J., Fu,S., Cao,X., Wu,H., Lu,J., Zeng,M., Liu,L., Yang,X. and Shen,Y. (2017) Synaptonemal complex protein 2 (SYCP2) mediates the association of the centromere with the synaptonemal complex. *Protein Cell*, **8**, 538–543.

2. Han,B.G., Nunomura,W., Takakuwa,Y., Mohandas,N. and Jap,B.K. (2000) Protein 4.1R core domain structure and insights into regulation of cytoskeletal organization. *Nat. Struct. Biol.*, **7**, 871–875.

3. Nunomura,W., Takakuwa,Y., Parra,M., Conboy,J. and Mohandas,N. (2000) Regulation of protein 4.1R, p55, and Glycophorin C ternary complex in human erythrocyte membrane. *J. Biol. Chem.*, 10.1074/jbc.M002492200.

4. Woltering,D., Baumgartner,B., Bagchi,S., Larkin,B., Loidl,J., Santos,T.D.L. and Hollingsworth,N.M. (2000) Recombination Checkpoint Functions Require Physical Interaction between the Chromosomal Proteins Red1p and Hop1p. *Mol. Cell. Biol.*, **20**, 6646–6658.

5. West,A.M.V., Komives,E.A. and Corbett,K.D. (2018) Conformational dynamics of the Hop1 HORMA domain reveal a common mechanism with the spindle checkpoint protein Mad2. *Nucleic Acids Res.*, **46**, 279–292.

6. Rosenberg,S.C. and Corbett,K.D. (2015) The multifaceted roles of the HOR MA domain in cellular signaling. *J. Cell Biol.*, **211**, 745–755.
